# Describing the landscape of nutrition- and diet-related randomized controlled trials: metaresearch study of protocols published between 2012 and 2022

**DOI:** 10.1016/j.ajcnut.2025.01.016

**Published:** 2025-01-24

**Authors:** Flávia Moraes Silva, Amanda Rodrigues Amorim Adegboye, Celeste Naude, Cintia Curioni, Fabio S Gomes, Gary S Collins, Gilberto Kac, Jennifer Anne de Beyer, Jonathan Cook, Leila Cheikh Ismail, Matthew J Page, Neha Khandpur, Sallie Lamb, Sally Hopewell, Shaima Saleh, Shona Kirtley, Simone Bernardes, Solange Durão, Colby J Vorland, Michael Maia Schlussel

**Affiliations:** 1 Nutrition Department and Graduate Program of Nutrition Science, Federal University of Health Science of Porto Alegre, Porto Alegre, Brazil; 2 Centre for Healthcare Research and School of Nursing, Midwifery and Health, Faculty of Health and Life Sciences, Coventry University, Centre for Agroecology, Water and Resilience, Coventry, United Kingdom; 3 Centre for Evidence-based Health Care, Department of Global Health, Faculty of Medicine and Health Sciences, Stellenbosch University, Cape Town, South Africa; 4 Department of Nutrition in Public Health, University of the State of Rio de Janeiro, Rio de Janeiro, Brazil; 5 Pan-American Health Organization, World Health Organization, Washington, DC, United States; 6 UK EQUATOR Centre, Centre for Statistics in Medicine, Nuffield Department of Orthopaedics, Rheumatology and Musculoskeletal Sciences, University of Oxford, Oxford, United Kingdom; 7 Nutritional Epidemiology Observatory, Department of Social and Applied Nutrition, Institute of Nutrition Josué de Castro, Federal University of Rio de Janeiro, Rio de Janeiro, Brazil; 8 Oxford Clinical Trials Research Unit, Centre for Statistics in Medicine, Nuffield Department of Orthopaedics, Rheumatology and Musculoskeletal Sciences, University of Oxford, Oxford, United Kingdom; 9 Department of Clinical Nutrition and Dietetics, College of Health Sciences, University of Sharjah, Sharjah, United Arab Emirates; 10 Nuffield Department of Women’s & Reproductive Health, University of Oxford, Oxford, United Kingdom; 11 Methods in Evidence Synthesis Unit, School of Public Health and Preventive Medicine, Monash University, Melbourne, VIC, Australia; 12 Division of Human Nutrition and Health, Wageningen University, the Netherlands; 13 Department of Nutrition, University of São Paulo, São Paulo, Brazil; 14 Department of Nutrition, Harvard T.H. Chan School of Public Health, Harvard, Boston, MA, United States; 15 Faculty of Health and Life Sciences, University of Exeter, Exeter, United Kingdom; 16 Health Systems Research Unit, South African Medical Research Council, South Africa; 17 Department of Applied Health Science, Indiana University School of Public Health-Bloomington, Bloomington, IN, United States

**Keywords:** nutrition, interventions, randomized controlled trial, protocols, research transparency, reporting guidelines, metaresearch

## Abstract

**Background:**

Publishing protocols promotes transparency and reproducibility. The scope and methods of protocols for nutrition- and diet-related randomized controlled trials (RCTs) have not been investigated yet.

**Objectives:**

This study aims to map the landscape of nutrition- and diet-related interventions research.

**Methods:**

We conducted a metaresearch of nutrition-and diet-related RCT protocols published between January 2012 and March 2022, in any language, targeting human participants, evaluating nutrition interventions isolated or combined. A systematic search of the literature was conducted in 6 online databases. Bibliometric information, study characteristics, and research transparency practices data were collected from the included publications. The instructions for authors of journals with publications in our sample were checked for endorsement of reporting guidelines. Mentions to reporting guidelines in the included protocols were also checked.

**Results:**

Among the 62,319 records retrieved, 1068 were eligible. The number of published protocols increased annually, with a mean of 103 (range: 32–163) publications/y. Protocols were published in 148 journals, 50 of them (33.8%) endorsed Standard Protocol Items: Recommendations for Interventional Trials (SPIRIT), 111 (75.3%) Consolidated Standards of Reporting Trials (CONSORT), and 4 (2.7%) Template for Intervention Description and Replication (TIDieR), whereas 343 (32.1%) protocols mentioned SPIRIT, 297 (27.8%) CONSORT, and 20 (1.9%) TIDieR. Most protocols reported the RCT registration number (*n* = 1006; 94.2%) and included statements about conflicts of interest (*n* = 952; 89.1%) and funding (*n* = 994; 93.2%). About two-thirds of protocols focused on adults or elderly participants (*n* = 677; 63.4%). Most protocols described 1 isolated nutrition- or diet-related intervention (*n* = 724; 67.8%), which were most frequently “supplementation, supplements or fortification” (*n* = 405; 37.9%) or “nutrition education, counseling or coordination of care” (*n* = 354; 33.1%). The most frequent primary outcomes reported were related to clinical status (*n* = 308; 28.8%).

**Conclusions:**

The number of protocols for nutrition- or diet-related RCTs published is increasing, supporting the raising awareness and the importance of promoting these publications. The support and mention of relevant reporting guidelines by journals and researchers, respectively, remain far from ideal.

## Introduction

Randomized controlled trials (RCTs) provide important evidence for clinical decision-making [1]. Making RCT protocols publicly available has been promoted as a good research practice as it increases research transparency and rigor [2]. Registration of RCTs is required by many research sponsors, funders, and journals in several countries and is recommended by the International Committee of Medical Journals [3]. However, also making the study protocol publicly available provides a more complete and detailed description of the planned research, compared with the limited templates offered by registration platforms [2,[4], [5], [6]].

Publicly available complete RCT protocols aligned with relevant reporting standards help to ensure consistency of trial procedures, ethical assumptions, transparency, and reliability of research findings [7]. Submitting a protocol for peer-reviewed publication early in the research pipeline potentially increases research quality, as it provides researchers with comments from external experts, aids with the interpretation of study results, and reduces selective outcome reporting [5,6].

Concerns about the quality and integrity of research published in the field of nutrition reflect those observed for other fields and reflect widespread concerns about a “credibility crisis” [8]. In response to this crisis, the scientific community has called for more rigor and transparency in the editorial process of scientific journals [7,9], including requests for detailed statements on conflicts of interests and funding, preregistration of study hypotheses and methods, and endorsement of reporting guidelines [9]. However, these practices are not yet universal. In several biomedical disciplines, including nutrition, <50% of journals endorse reporting guidelines [9,10].

To the best of our knowledge, no study has investigated the scope and methods described in protocols of nutrition- and diet-related RCTs or how often they are published. We aimed to map the contemporary landscape of nutrition- and diet-related interventions research based on RCT protocols published between 2012 and 2022. We also aimed to investigate the appearance of research transparency and reproducibility practices in these publications.

## Methods

### Study design

This metaresearch (a study of research itself—its methods, reporting and reproducibility) [11] was registered on the Open Science Framework (registration: https://doi.org/10.17605/OSF.IO/YWEVS) and the full protocol was published [12]. It followed the methodology of a systematic review for literature search, screening of eligible protocols and data extraction. We extracted data from protocols of nutrition- and diet-related RCTs published as scientific articles.

We searched for relevant protocols on PubMed, Embase, CINAHL, Web of Science, PsycINFO, and the Global Health Database between 1 January, 2012 and 24 March, 2022.

### Eligibility criteria

We included RCT protocols if: *1*) study design was self-identified by the trialists as an RCT (that is, whether the authors described their studies as RCTs); *2*) aimed to include humans as participants, regardless of age, nutritional status and clinical condition; *3*) aimed to evaluate any outcome; *4*) was published in any language; and *5*) aimed to evaluate ≥1 nutrition or diet-related intervention isolated or combined with other interventions (such as exercise or drugs) or as part of a lifestyle or health program intervention.

We included 5 broad categories of intervention: *1*) diets, dietary components, and dietary patterns; *2*) formulated, fortified, and enriched foods; *3*) dietary products, including dietary supplements; *4*) nutrients and bioactive non-nutrient components naturally present in foods (for example, cinnamon); and *5*) nutritional education, promotion, counseling, or programs [13].

We excluded protocols of RCTs if: *1*) only used pharmaceutical or herbal medicines as intervention, *2*) they were protocols of non-randomized trials, and *3*) if the publications reported also study findings.

### Literature search

The lead author (FMS) and a professional health sciences information specialist (SK) built a search strategy for PubMed (via the National Library of Medicine) combining the search strategy developed by Durão et al. to identify diet and nutrition trials [14] and a modified version of the search strategy developed by Madden et al. to identify RCT protocols [15]. We adapted the search strategy to Embase (via Elsevier), CINAHL (via EBSCO), Web of Science (via Clarivate), PsycINFO (via Ovid), and Global Health Database (via Ovid). We ran the search strategies for all databases on 24 March, 2022 (see complete search strategy in Supplemental Box 1).

### Selection of eligible reports

We imported all retrieved references into EndNote (21.0, Clarivate Analytics) and used its automated deduplication feature to remove duplicates. We exported the records to the web and mobile app Rayyan [16]. The lead author (FMS) manually double-checked the resulting reference list and removed the remaining duplicates. Two reviewers (FMS and JL) independently screened the publications’ titles and abstracts to check for eligibility. One reviewer (FMS) then screened potentially eligible full texts. Disagreements between reviewers were resolved by consensus.

### Data collection

One reviewer (FMS) extracted data using a standardized data extraction form in REDCap®, v9.1.0—Vanderbilt University [17] and it can be accessed in the Supplemental Box 2. In a sample of 100 protocols, another reviewer (SS) also extracted all data from our data extraction form and the concordance rate was calculated and presented a mean equal to 96.5%, ranging from 89.1% to 100%. Our research team considered the concordance rate acceptable, and 1 reviewer (FMS) followed with the data extraction of the remaining protocols.

We collected the first author’s name, journal, year of publication, bibliometric information, research transparency practices (for example, funding and conflicts of interest statements (yes/no) and details of protocol registration), and general study characteristics using the participants, interventions, comparators, outcomes, and study designs (PICOS) format, as detailed in Table 1.

**TABLE 1:** PICOS categories used for data extraction and characterization of the nutrition- and diet-related protocols’ scope.

Categories used for data extraction	
Participants	• Pregnant women • Mother and infant pairs • Infants • Children and preschool-aged children • Adolescents • Adults (18–65 y) • Elderly (≥65 y) • Adults and elderly (≥18 y) • Families • Postmenopausal women • Participants with a clinical condition^1^
Interventions^2^	• Food (whole food, food products, specially formulated foods) • Lactation, complementary feeding • Complete diet or dietary pattern • Complete nutrition formulas (enteral or parenteral) • Supplementation, or supplements, or fortification (single or multiple nutrients, bioactive non-nutrients, plant components) • Nutrition education, counseling, and coordination of care • Other, if no component of intervention could be categorized as any of the above
Comparator	• Placebo • No intervention • Usual care • Different intervention • Other
Outcomes	• Mortality • Clinical status (clinical or biochemical measures) • Nutritional status (anthropometry, body composition, nutrition diagnosis) • Frequency or severity of disease • Diet quality and/or variety • Food/ nutrient/dietary intake • Diet-related behaviors • Other non-dietary behaviors • Withdrawal from the study, drop-out or adherence related • Adverse events, side-effects and/or safety • Cost-effectiveness or economic • Quality of life • Other
RCT design	• Parallel • Crossover • Cluster • Factorial • Pilot • Multicenter/ Single center
Study framework	• Superiority • Equivalence • Non-inferiority • Exploratory • Not reported

Abbreviations: PICOS, participants, interventions, comparators, outcomes, and study designs; RCT, randomized clinical trials.

1 Participants’ clinical conditions were grouped according to the type of disease: endocrine, lung, cardiovascular, gastrointestinal, infectious, muscle/skeletal/rheumatological, psychiatric diseases, kidney, neurological, gynecological, and others (individual frequency ≤1.0%).

2 The nutrition intervention categories of interest were adapted from Naude et al. [13]. We classified as “isolated nutrition intervention” the published RCT protocols that aimed to test the effect of 1 nutrition intervention category of interest not combined with other interventions such as exercise, drugs, medical care, and meditation. The planned period within which the intervention would be delivered (different from follow-up period) was extracted from the protocols. Interventions in the category “complete diet or dietary pattern” were grouped by type of diet: Mediterranean, low-carb, low-fat, healthy (as defined by the authors, based on a specific dietary guideline or on the macronutrients proportion of daily energy), personalized, intermittent fasting, Dietary Approach for Stop Hypertension or sodium-restricted, high-quality carb, and others. Interventions in the category “supplementation, supplements, or fortification” were grouped by the type of supplement: vitamins, minerals, probiotics and symbiotic, carbohydrates and fiber, fats, protein and amino acids, bioactive components, and others. Protocols were categorized according to the duration of the intervention: fixed (if the authors described a unique period for all participants), not fixed (if the duration of the intervention depended on the incidence of outcomes and was not the same for all participants), and not reported. Interventions were classified as ‘acute response’ if the outcomes were evaluated 24 h after delivering the intervention.

Reporting of funding and conflicts of interest were categorized as: no reporting of funding/ no reporting of conflicts of interest, reporting of no funding/ reporting of no conflicts of interest, and reporting of funding/reporting of conflicts of interest. We did not investigate the type of funding, the funders’ role in the study conduction, or the type of conflicts of interest.

Journals were categorized as medical or health-related, methods, or nutrition journals based on their scope checked in the webpages. One reviewer (SB) collected data on journals’ endorsement of the “Standard Protocol Items: Recommendations for Interventional Trials” (SPIRIT) [6], CONSORT [18], and “Template for Intervention Description and Replication” (TIDieR) [19] reporting guidelines for writing up protocols of trials, trials, and interventions, respectively. The reviewer screened the instructions for the authors webpages of each journal identified in our sample. Endorsement was characterized by a general recommendation to follow the relevant reporting guidelines or a requirement that authors should adhere to the relevant reporting guidelines’ checklists when writing their manuscripts, regardless of whether the complete checklists should be submitted or not. One reviewer (FMS) checked whether authors mentioned these reporting guidelines in their papers (that is, self-reported adherence to reporting guidelines or formal citation). Thus, “endorsement of reporting guidelines” was related to the journal where the protocols were published whereas the “mention of reporting guidelines” was related to the information provided by the authors of protocols in the manuscripts.

### Amendments to the protocol

The original data extraction form was published alongside our protocol [12]. We added 8 questions to this form: *1*) Is it a pilot study? (yes, no); *2*) What is the framework of the RCT? (superiority, equivalence, non-inferiority, exploratory, not reported); *3*) What was the country where the RCT is being planned? *4*) Was the RCT registered? (yes, no). If so, where? (registration platform name); *5*) What are the details of the intervention?; *6*) What is the intervention duration (in days), if delivered for a fixed period?; *7*) Is there a declaration of conflicts of interest in the manuscript?; *8*) Is there a funding statement in the manuscript? The final data extraction form is available as Supplemental material to this article (Supplemental Box 2).

We also added the evaluation of journal endorsement of reporting guidelines, and we collected the journals’ 2021 impact factor from Web of Science.

### Data analysis

The frequency of each PICOS component and reporting transparency practices was calculated by year of publication, geographical location of nutrition and diet-related RCTs (focusing on the 5 most common countries), and the subgroups of protocols involving patients with cancer and cardiovascular diseases, as these are the major global causes of death [20].

The statistical package SPSS 22.0 was used for data tabulation and analyses. We calculated the absolute and relative frequency of all categorical variables and presented the results as *n* (%). We present medians and ranges (minimum–maximum) for quantitative variables. Graphics were designed in Excel.

## Results

### Literature search and selection of published RCT protocols

**FIGURE 1:**
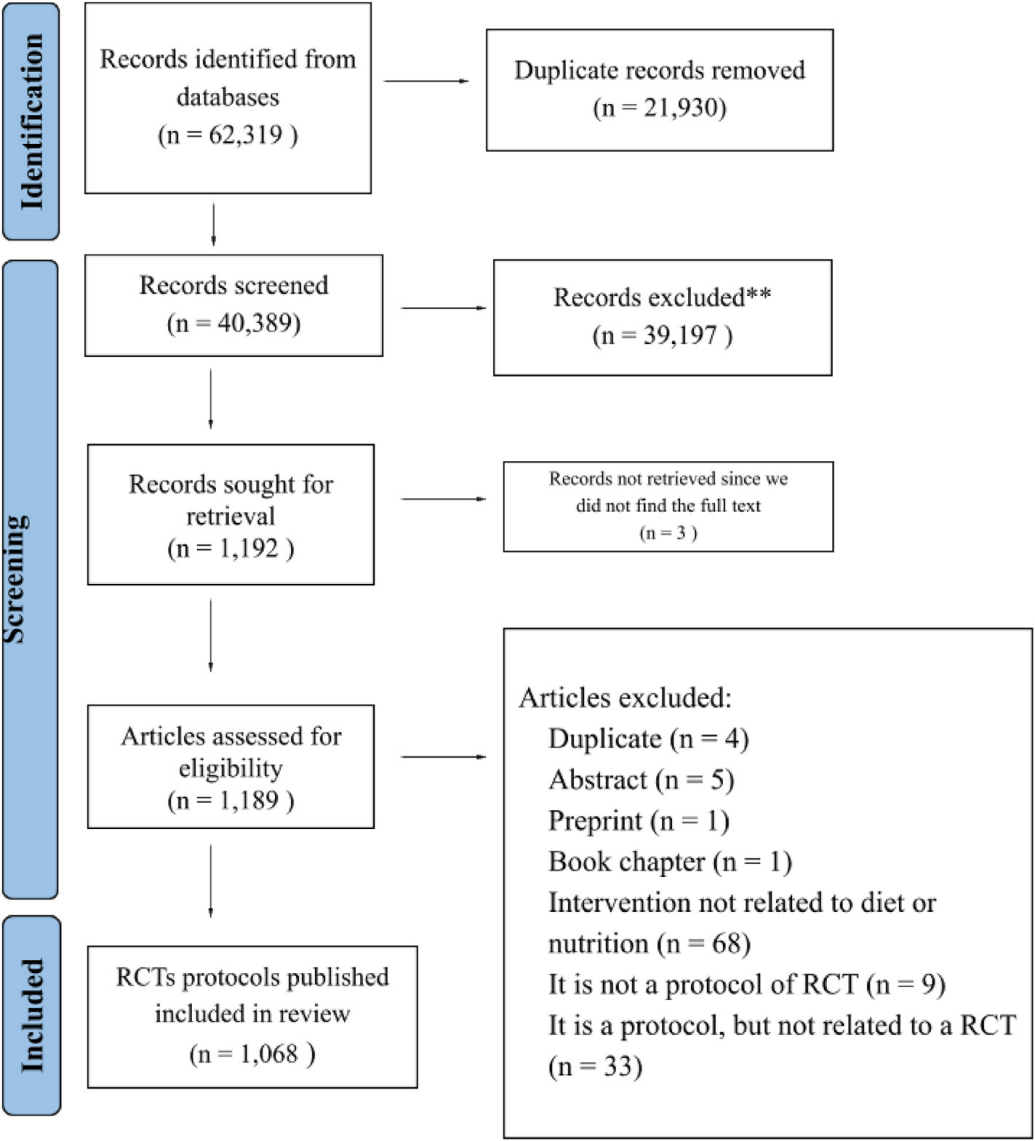
Flow chart of the selection of nutrition- and diet-related RCT protocols, published 2012–2022. RCT, randomized controlled trials. ∗∗Records excluded for not meeting the eligibility criteria, as evidenced by screening their titles and abstracts.

The literature search retrieved 62,319 records. We screened the titles and abstracts of 40,389 of them, after removing 21,930 duplicates. We screened the full texts of 1189 articles (because 3 articles potentially eligible could not be retrieved), excluding 121 publications. Protocols of 1068 RCTs met inclusion criteria and were included in this metaresearch study (reference list available as a Supplemental Excel file named as “reference list”). Figure 1 summarizes the detailed selection process.

### General characteristics of the published RCT protocols

**FIGURE 2:**
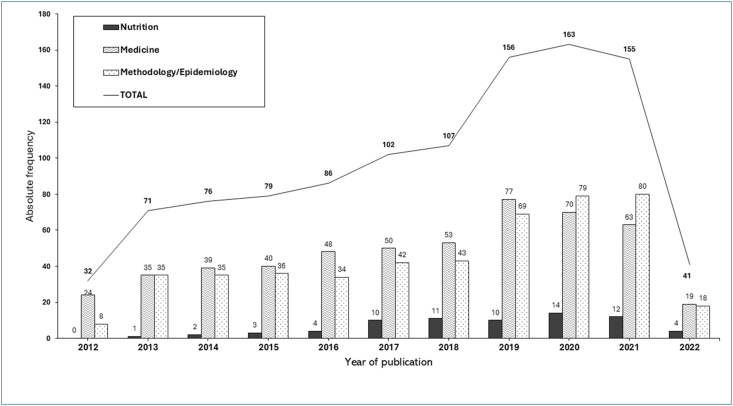
Number of nutrition- and diet-related RCT protocols published between 2012 and 2022 by year and journal scope (∗corresponding to the first 3 mo of 2022). RCT, randomized controlled trials.

**FIGURE 3:**
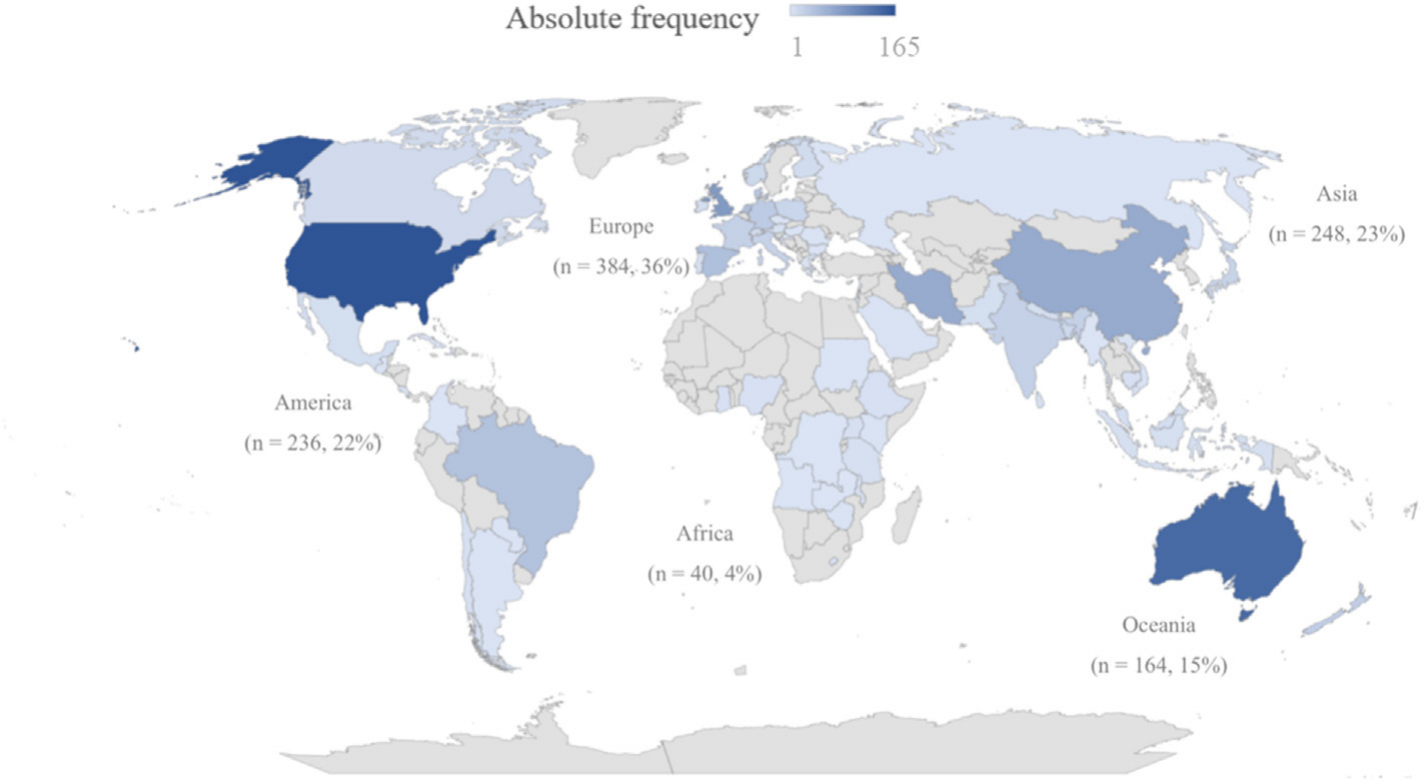
Geographical distribution of nutrition- and diet-related RCT protocols published between 2012 and 2022. Absolute (relative) frequency given by continent. Intensity of shading indicates absolute frequency by country. RCT, randomized controlled trials.

The number of published nutrition- and diet-related RCT protocols increased annually between 2012 and 2021, with a mean of 103 (range: 32–163) publications/y and 41 protocols published in the first 3 mo of 2022 (Figure 2). The countries publishing the most protocols were the United States (*n* = 165; 15.5%), Australia (*n* = 137; 12.8%), United Kingdom (*n* = 72; 6.8%), Iran (*n* = 65; 6.1%), and China (*n* = 65; 6.1%). As illustrated in Figure 3, most protocols were published mainly in European countries (*n* = 384, 36%).

Most protocols (*n* = 1006; 94.2%) reported that the trial was registered. Clinicaltrials.gov (*n* = 520; 48.7%) was the most used registration platform, followed by the Australian and New Zealand Clinical Trial Register (*n* = 154; 14.4%), and the International Standard Randomized Clinical Trial register (*n* = 117; 11.0%). Most published protocols included a statement about conflicts of interest (*n* = 952; 89.1%), of which 783 (82.3%) declared no conflicts of interest. More than 90% of the publications included a funding statement (*n* = 994; 93.2%). Only 48 (4.5%) published protocols declared that the RCT was not funded.

The protocols were published in 148 journals, 114 (77.0%) of them from the medical scientific research field, 21 from nutrition (14.2%) and 13 (8.8%) from the methods scientific research field. Figure 2 shows the absolute frequency of protocols published by year according to the journal category. Few protocols were published in nutrition journals (*n* = 71; 6.6%), with the rest split between medical journals (*n* = 518; 48.5%) and methods journals (*n* = 479; 44.9%). The most used journals were *Trials*, a methods journal (*n* = 295; 27.6%), *BMJ Open*, a medical journal (*n* = 152,14.2%), and *Contemporary Clinical Trials*, a methods journal (*n* = 103; 9.8%) (Table 2). Forty-four journals did not have an impact factor. The rest had impact factors ranging from 0.813 to 20.999.

**TABLE 2:** Journals in which protocols of nutrition- and diet-related RCTs were more frequently published (*n* = 1068 protocols published between 2012 and 2022).

Journal	Impact factor^1^	Scientific field	Frequency of protocols, *n* (%)^2^
*Trials*	2.728	Methods	295 (27.6)
*BMJ Open*	3.007	Medicine	152 (14.2)
*Contemporary Clinical Trials*	2.261	Methods	103 (9.8)
*BMC Public Health*	4.125	Medicine	101 (9.5)
*JMIR Research Protocols*	Not identified^1^	Methods	30 (2.8)
*BMC Pregnancy and Childbirth*	3.105	Medicine	26 (2.4)
*BMC Paediatrics*	2.922	Medicine	26 (2.4)
*Medicine Open*	Not identified^1^	Medicine	23 (2.3)
*BMC Cancer*	4.638	Medicine	20 (1.9)
*Nutrition Journal*	4.344	Nutrition	17 (1.6)
*Pilot and Feasibility Studies*	Not identified^1^	Methods	16 (1.5)
*BMC Geriatrics*	4.070	Medicine	13 (1.2)
*Nutrients*	6.706	Nutrition	13 (1.2)
*Others*^3^	0.813–20.999	Medicine	160 (15)
		Methods	35 (3.3)
		Nutrition	38 (3.6)

Abbreviation: RCT, randomized controlled trial.

1 Fourty-four journals did not have an impact factor.

2 Journals with ≤1% of all protocols were grouped.

3 Total protocols published between 2012 and 2022.

We examined the 148 journals’ instructions for authors’ pages. Ninety-five (64.2%) journals endorsed reporting guidelines in general, recommending that authors search for reporting guidelines on the EQUATOR Network website. Fifty (33.8%) journals endorsed SPIRIT, 111 (75.3%) endorsed CONSORT, 4 (2.7%) endorsed TIDieR, and 1 of the 4 explicitly endorsed TIDieR’s use in protocols. Fifty-four (36.5%) journals required authors to submit the relevant reporting guideline’s checklist alongside their manuscript.

**FIGURE 4:**
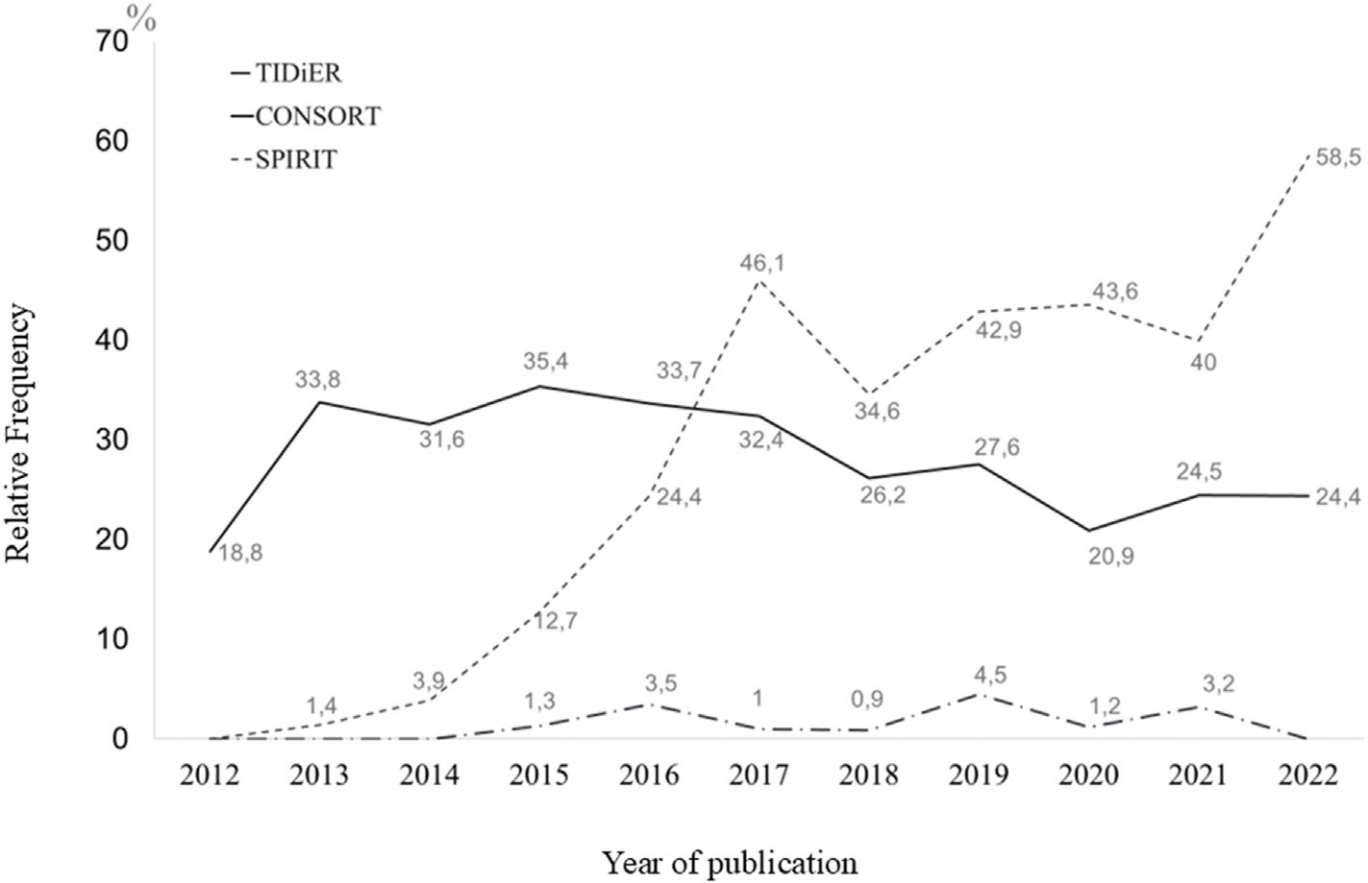
Relative frequency of nutrition- and diet-related RCT protocols published between 2012 and 2022 that referenced SPIRIT, CONSORT, and TIDieR. RCT, randomized controlled trials; SPIRIT, Standard Protocol Items: Recommendations for Interventional Trials; TIDieR, Template for Intervention Description and Replication.

We also examined whether the protocols cited or mentioned reporting guidelines. SPIRIT was cited or mentioned in 343 (32.1%) protocols, CONSORT in 297 (27.8%) protocols, and TIDieR in 20 (1.9%) protocols. The proportion of protocols mentioning reporting guidelines did not increase linearly with time (Figure 4). For example, mentions of CONSORT ranged from a low of 18.8% in 2012 to a high of 35.4% in 2015. Mentions of TIDieR ranged from a low 0% in 2012 to 2014 (when TIDieR was published) to a peak of 4.5% in 2019, dropping again to 3.2% in 2021. Supplemental Figure 1 shows the relative frequency of nutrition- and diet-related RCT protocols published 2012–2022 that referenced the CONSORT, SPIRIT, and TIDieR reporting guidelines, grouped by type of journal: it was similar between journals for CONSORT and TIDieR whereas the reference of SPIRIT was higher in methods’ journals. Supplemental Table 1 presents the proportion of protocols of nutrition- and diet-related RCTs published 2012–2022 that mention the CONSORT, SPIRIT, and TIDieR reporting guidelines, by whether the journal the protocol is published in endorses that reporting guideline. Most protocols published in journals that endorse the SPIRIT (45.4% of journals) and CONSORT (95.1% of journals) did not mention these reporting guidelines in the manuscript: no mention of SPIRIT and CONSORT in 74.6% and 71.3%, respectively. Only 7 journals endorse the TIDieR, but no protocol mentions it. Among the protocols published in journals without endorsement of SPIRIT (54.6% of journals) and CONSORT (4.9% of journals), 37.7% mentioned SPIRIT in the manuscript whereas 9.6% mentioned CONSORT.

### PICOS components of the nutrition- and diet-related published RCT protocols

Table 3 characterizes the scope of the protocols using their PICOS. Most protocols described target populations of adults and the elderly (*n* = 350; 32.7%) or only adults (*n* = 252; 23.6%). About one-third of the protocols included healthy individuals (*n* = 342; 32.0%), 21.4% (*n* = 229) included participants with endocrine diseases, and 8.4% (*n* = 90) included participants with cardiovascular diseases.

**TABLE 3:** PICOS details used in nutrition- and diet-related RCT protocols published 2012–2022.

Participants			
Categories of participants	Number (%)	Clinical conditions of participants	Number (%)
Adults and elderly	350 (32.7)	None (healthy)	342 (32)
Adults	252 (23.6)	Endocrine	229 (21.4)
Children	132 (12.3)	Cardiovascular	90 (8.4)
Pregnant women	99 (9.3)	Gastrointestinal/hepatic	82 (7.7)
Elderly	76 (7.1)	Muscle/skeletal	49 (4.6)
Infants	66 (6.2)	Infectious	39 (3.7)
Adolescents	63 (5.9)	Psychiatric	31 (2.9)
Families	53 (5.3)	Critically ill	28 (2.6)
Mother and infants	19 (1.8)	Kidney	27 (2.5)
Postmenopausal women	9 (0.8)	Neurological	25 (2.3)
		Malnutrition	24 (2.2)
		Gynecological	23 (2.2)
		Lung	20 (1.9)
		Others	59 (5.5)
Intervention			

Type of the RCT intervention	Number (%)	Category of nutritional intervention	Number (%)
Isolated nutritional intervention	724 (67.8)	Supplementation	
Nutritional intervention as a component of a lifestyle intervention	127 (11.9)	Supplementation, or supplements, or fortification	405 (37.9)
Nutritional intervention combined with exercise	98 (9.2)	Nutrition education, counseling, and coordination of care	354 (33.1)
Nutritional intervention as a component of a health program	83 (7.8)	Complete diet or dietary pattern	165 (15.4)
Nutritional intervention combined with drugs	18 (1.7)	Food (whole food, food products, specially formulated foods)	78 (7.3)
Nutritional intervention combined with other type of medical care	18 (1.7)	Complete nutrition formulas (enteral or parenteral)	37 (3.5)
		Lactation, complementary feeding	17 (1.6)
		Others	12 (1.1)
Comparators			

Type	Number (%)	Total of arms	Number (%)
Placebo	362 (33.9)	2	844 (79.1)
Usual care	316 (29.6)	3	134 (12.5)
Other intervention	289 (27.1)	4	75 (7.0)
No control	101 (9.5)	5 or more	15 (1.4)

Outcomes			

Primary outcomes categories	Number (%)	Primary outcomes categories	Number (%)
Clinical status	308 (28.8)	Mortality	35 (3.3)
Nutritional status	247 (23.1)	Quality of life	26 (2.4)
Frequency or severity of disease	238 (22.3)	Breastfeeding	19 (1.8)
Food/nutrient/dietary intake	68 (6.4)	Diet quality and/or variety	17 (1.6)
Functional status	57 (5.3)	Adverse events, side-effects	7 (0.7)
Withdrawal from the study, drop-out	56 (5.2)	Cost-effectiveness or economic	2 (0.2)
Other non-dietary behaviors	44 (4.1)	Other	131 (12.3)
Diet-related behaviors	39 (3.7)		

Study design and framework			

Design	Number (%)	Framework	Number (%)
Parallel	1014 (94.9)	Superiority	755 (70.7)
Crossover	54 (5.1)	Exploratory	92 (8.6)
Cluster	138 (12.9)	Non-inferiority	20 (1.9)
Pilot	72 (6.7)	Equivalence	7 (0.7)
Factorial	39 (3.7)	Not reported	194 (18.2)
Multicentric	230 (19.3)		

Abbreviations: PICOS, participants, interventions, comparators, outcomes, and study designs; RCT, randomized controlled trial.

Categories within each of the PICOS components are not mutually exclusive.

The most common aim described in the protocols was to estimate the effect of an isolated nutrition or diet-related intervention (*n* = 724; 67.8%) whereas in 127 (11.9%) published protocols the nutrition intervention was part of a lifestyle intervention and in 83 protocols (7.8%) it was part of a health program intervention. In the remaining protocols, the nutrition or diet-related intervention was combined with exercise (*n* = 98, 9.2%), drugs (*n* = 18, 1.7%) or other medical intervention (*n* = 18, 1.7%). “Supplementation, supplements, or fortification” (*n* = 405; 37.9%) and “nutrition education, counseling, or coordination care” (*n* = 354; 33.1%) were the most common types of interventions studied. The effect of a specific diet or dietary pattern was to be evaluated in 165 protocols (15.4%). Only 101 (9.4%) protocols included no intervention (such as a waiting list) in the control group whereas the most frequent comparators were placebo (33.9%) and usual care (29.6%).

Among the protocols with “supplementation, supplements, or fortification” interventions, vitamins (*n* = 126; 31.1%), probiotics (*n* = 63; 15.6%), and minerals (*n* = 50; 12.3%) were most frequently used (Supplemental Figure 2A). These protocols mostly used vitamin D (*n* = 76; 60.3%) and the mineral iron (*n* = 16; 32.0%). Among the protocols proposing to evaluate the effect of a specific diet or dietary pattern, the Mediterranean diet (*n* = 26; 15.7%), low-carb diet (including ketogenic and Paleolithic diets) (*n* = 22; 13.3%), and energy-restricted diet (*n* = 19; 11.5%) were the most frequently chosen (Supplemental Figure 2B).

Thirty-eight protocols (3.6%) planned to evaluate the response of an intervention within 24 h of exposure. Most protocols were proposed to evaluate the response to the intervention after longer periods (*n* = 899; 84.2%), with a median period under the active intervention of 120 (minimum 2; maximum 2160) d. The period under the active intervention was not fixed and relied on outcome incidence in 121 protocols (11.3%). The remaining 10 (0.9%) protocols did not report information about the time under the intervention.

The most frequent primary outcomes reported by the protocols were “clinical status” (*n* = 308; 28.8%), “nutritional status” (*n* = 247; 23.1%), and “frequency or severity of disease” (*n* = 238; 22.3%). Most protocols described a single-center study (*n* = 838; 78.5%) with a 2-arm (*n* = 844; 79.1%), parallel (*n* = 1014; 94.9%) design and a superiority framework (*n* = 755; 70.7%).

Characteristics of protocols according to the year of publication, country that the trial would take place in, and whether participants had a cancer or cardiovascular disease diagnosis can be found in the Supplemental Results (Supplemental Figures 3–4 and Supplemental Tables 2–4).

## Discussion

In this metaresearch study, we evaluated 1068 protocols for nutrition- and diet-related trials published in journals indexed in 6 online databases of medical literature between 2012 and 2022. The protocols mostly tested supplementation interventions and aimed to investigate their effects on indicators of clinical outcomes in adults and the elderly with a disease. Most protocols were published in general medical and health-related journals or methods journals and included protocol registration information, and statements declaring conflicts of interest and funding sources. Few protocols mentioned a relevant reporting guideline.

Our results are consistent with those of a cross-sectional study on the scope and quality of Cochrane reviews of nutrition interventions published between 2007 and 2015, which also found supplementation to be the most frequently studied intervention (50%) and clinical or nutritional status assessment of the most frequently evaluated primary outcomes (82.1%) [13]. “Supplementation, supplements, or fortification” was the most frequently used intervention category in 2016–2018 and 2021–2022. During the rest of the period covered by the study, most protocols aimed to evaluate the effect of a “nutrition education, counseling, and coordination care” intervention. Protocols of RCTs with “Supplementation, supplements, or fortification” interventions might be more common because these are generally easier to deliver and require fewer behavioral modifications from participants than “complete diet or dietary pattern” and “nutrition education, counseling, and coordination of care” interventions [13,21]. More than 70% of protocols used outcomes related to clinical status, nutritional status, and frequency or severity of disease, regardless of the publication year. These outcomes are achievable in the short term, matching the median intervention duration of 120 d.

Most of the protocols were registered on a clinical trial registration platform. Since the late 1990s, trial registration has been required by law in some countries [22]. Study registration is considered a good research practice because it can reduce publication and hindsight bias, safeguard honest research, and minimize research waste. A public registration record enables verification that the content of the research report corresponds to what was planned, particularly when a detailed protocol is not publicly available [[23], [24], [25]].

The absolute number of protocols of nutrition- and diet-related RCTs published as scientific articles increased between 2012 and 2021. However, we estimate that despite this increase the proportion of nutrition RCTs that had a protocol published remained low during the period (Supplemental Discussion, Supplemental Table 5). Published RCT protocols contribute to increased transparency and robustness of research methods and findings, as these articles can give much more detail than study registration entries [26,27]. Although journals increasingly support and publish RCT protocols, the low adoption of this practice is also observed in other fields of medical research [28]. A metaresearch study of 326 RCTs (63.5% drug trials) found that only 36.2% made their protocols publicly available, mostly as peer-reviewed publications (47.5%) or Supplemental files with the primary results (40.7%) [4]. Thus, awareness about the benefits of this research practice still needs to be raised, both in the field of nutrition interventions and general health care research.

We found that few of the protocols in our sample were published in nutrition journals, as most were published in general medical or methods journals. Protocols are still outside the scope of several nutrition journals, showing that more of these journals should allow and incentivize the publication of these important articles as an approach to promote transparency and reproducibility and ensure good practices for the conduction of human nutrition RCTs. These practices need to be planned during the protocol development stage and involve documentation and regulation of RCTs [29], planning and conducting statistical analyses [30], as well as adopting the best practices for data management [31]. These findings also highlight the importance of engaging with stakeholders from the wider scientific community, as interest in publishing nutrition- and diet-related interventions research clearly exists beyond the nutrition research community, so our message should not be restricted to our peers.

Greater transparency in disclosing all potential conflicts of interest can help stakeholders better understand who is proposing which research questions and the motivations behind studies [32]. An analysis of 2,751,420 open-access records on PubMed Central showed that some reporting transparency indicators were increasingly met between 2000 and 2020, including disclosure of conflicts of interest. For instance, the proportion of research articles reporting funding and conflicts of interest increased from 25% and 0% in 2000 to 89% and 91% in 2020, respectively [33]. While most protocols in our study reported conflicts of interest and funding statements, these statements tended to be short and vague, providing little or no information about potential conflicts beyond financial conflicts. Indirect financial benefits and non-financial conflicts of interest can also influence research outcomes and should be disclosed.

The protocols analyzed were published in 148 journals, of which 75.3% endorsed CONSORT, 33.8% endorsed SPIRIT, and 2.7% endorsed TIDieR in their instructions for authors. A metaepidemiological study found that only 90 (53%) out of 170 endocrine and internal medicine journals supported the CONSORT statement, with rates per specialty ranging from 9% in hematology journals to 63% in internal medicine journals [10]. Another study examined editorial procedures to improve the reporting of empirical studies in nutrition and dietetics research and showed that 27/30 (90%) of journals with high impact factors mentioned CONSORT and 7/30 (23.3%) mentioned SPIRIT in the instructions for authors [9]. These differences might be due to the evolution of journal endorsement of reporting guidelines in the last decade and the scientific field, since studies published in 2018 showed varying frequencies of CONSORT endorsement among the journals related to cardiology (5% of 19), critical care (14% of 37), dermatology (30% of 20) and oncology (52% of 21). So, endorsement of reporting guidelines remains suboptimal. Journals can play an essential role in improving transparency in research reporting, as their endorsements indicate to authors the degree of completeness expected from them in their publications [34].

Journal endorsement of reporting guidelines and author citation of reporting guidelines do not guarantee adherence by authors. The increase in the number of protocols mentioning SPIRIT does not necessarily mean these protocols reported all the information required by SPIRIT. Similarly, we cannot assume that a publication is not complete and transparent because a relevant reporting guideline was not mentioned in the text. Nevertheless, the discrepancy between the proportion of journals publishing nutrition RCT protocols that endorse SPIRIT, and the number of nutrition RCT protocols mentioning this reporting guideline highlights that awareness about this important tool should be increased among researchers in the field.

Our next step is to assess reporting completeness in a subsample of the protocols described here, as part of a research program to produce official developments for the CONSORT, SPIRIT, and PRISMA statements focusing on nutritional interventions [35]. This work is in line with the ongoing initiative of the Federation of European Nutrition Societies (FENS) to improve standards in the science of nutrition [36]. We are in close contact with the FENS working group to gather expert input, increase dissemination of the final recommendations, and ensure a consistent message is presented.

### Study limitations and future research

We aimed to describe the landscape of nutrition- and diet-related interventions research, based on a sample of RCT protocols published in indexed medical journals during a period of 10 y (between 2012 and 2022). One limitation of this study is that the protocols of many RCTs may never be published as articles [6]. However, our sample of publications consisted of protocols published in journals indexed in 6 online databases of medical research over a period of 10 y. Our findings on the main aspects of the protocols scope were similar to those previously described in the literature [13]. Although we have not included protocols published in the last 2 y, the trends of this type of publication appear not to have changed. We are therefore reasonably confident that this work provides a good representation of contemporary nutrition- and diet-related intervention research.

A steady increase in the number of records retrieved with our search strategies for both nutrition RCTs and nutrition RCT protocols was observed in the period, as can be seen in the Supplemental Table 5. We did not check whether the publications describing the results of the nutrition RCTs that had their protocols assessed in our study are available. Therefore, we cannot estimate the proportion of undisclosed RCTs in the field. Likewise, we could not assess consistency between the methods described in the protocols and in their respective RCT results publications. Future research could focus on these research questions.

We only performed a cross-sectional assessment of current journal endorsement of reporting guidelines, which is likely to have changed over the study period of 2012 to 2022. We might have missed important improvements in the endorsement of reporting guidelines by the journals in which the included protocols were published. It was also not the aim of our study to assess the methodological quality of nutrition RCT protocols because, as far as we know, there is no specific tool for this purpose. Future metaresearch could also focus on exploring this aspect of published nutrition RCT protocols. Also, they should explore the disclosure of funding sources and conflicts of interest in more detail, as these statements can play a role in the transparency and reproducibility of nutrition- and diet-related trials. Future research could investigate whether these practices are associated with reporting completeness and risk of bias in RCTs and their protocols.

## Conclusions

In conclusion, protocols describing nutrition- and diet-related RCTs are increasingly being published. Awareness of relevant reporting guidelines and their endorsement by journals remains far from ideal in the field, potentially hampering the publication of RCT protocols as a mechanism of research transparency and integrity. Most protocols of nutrition- and diet-related RCTs were not published in nutrition journals, underscoring the need to engage the editorial board of these journals to allow and incentivize this type of publication as a practice of transparency and reproducibility. Our findings can be used by researchers, institutions, and funders to assess the most studied populations, interventions, and outcomes in the field of nutritional intervention research and the most frequent study designs used to address these research questions and to identify areas for future research.

## Author contributions

The authors’ responsibilities were as follows – MMS, SK, SH, GK, GSC: jointly conceived the idea of this project; MMS, FMS, SL, FSG, JC, SK, SH, SD, CC, CN, JAdB, ARAA, MJP, NK, LCI, CJV, GK, GSC: contributed to the study design and development of research questions; FMS, SK: built the search strategy for all databases and performed them; FMS, MMS, GSC: designed the data extraction form; FMS, SB, SS: responsible for the data collection; FMS, MMS: analyzed the data; FMS, MMS: led the writing and were responsible for the final content of the manuscript; FSG: is a staff member of the Pan American Health Organization; and all authors: provided detailed comments on earlier drafts of the manuscript; have read and approved the final version of the manuscript, are responsible for the views expressed in this publication, and do not necessarily represent the decisions or policies of the Pan American Health Organization or their host institutions.

## Funding

The authors reported no funding received for this study.

### Data availability

Extracted data are available at OSF (https://doi.org/10.17605/OSF.IO/YWEVS) and the analysis code is available at OSF (https://doi.org/10.17605/OSF.IO/YWEVS).

## Conflict of interest

FMS received a postdoctoral fellowship from COPPETEC Foundation for the conduct of this work. MMS, SK, JAdB, and GC were funded by Cancer Research UK (grant C49297/A27294) at the time this research took place. MJP is supported by an Australian Research Council Discovery Early Career Researcher Award (DE200101618). CJV has received honoraria from The Obesity Society and his University has received funds to support his research from: National Cattlemen’s Beef Association; Alliance for Potato Research and Education; the Gordon and Betty Moore Foundation; and NIH.
