# Supplementary material for: Describing the landscape of nutrition- and diet-related randomized controlled trials: metaresearch study of protocols published between 2012 and 2022
Source: Am J Clin Nutr. 2025 Jan 24;121(4):882–91. doi: 10.1016/j.ajcnut.2025.01.016 (PMC12002212; doi:10.1016/j.ajcnut.2025.01.016)
Supplement: Multimedia component 1 [file mmc1.pdf]

Supplementary Material

Describing the landscape of nutrition- and diet-related randomised controlled trials: meta-research study of protocols published between 2012 and 2022

Authors

Flávia Moraes Silva, Amanda Rodrigues Amorim Adegboye, Celeste Naude, Cintia Curioni, Fabio S Gomes, Gary S Collins, Gilberto Kac, Jennifer Anne de Beyer, Jonathan Cook, Leila Cheikh Ismail, Matthew J Page, Neha Khandpur, Sarah Sallie Lamb, Sally Hopewell, Shaima Saleh, Shona Kirtley, Simone Bernardes, Solange Durão, Colby J Vorland, Michael Schlussel

Supplementary results

Characteristics of protocols according to the year of publication, country, and whether participants have a cancer or cardiovascular diagnosis

From 2012 to 2022, there was no discernible trend in the proportion of protocols according to participant life stage. Adults and the elderly were the most frequent categories in all years except 2012, when adult participants were more common. Most protocols focused on individuals with a specific clinical condition in all years. We found no clear relationship between intervention type and the year of protocol publication (**Supplementary Figure 3**). The primary outcomes used most often were markers of nutritional status in 2012 and 2013, indicators of frequency or severity of disease in 2015, and clinical status in all other years. Most protocols described two-arm parallel RCTs, regardless of year. The lowest frequencies of protocol registration (85.9%) and conflicts of interest disclosure (81.7%) were observed in 2013. The frequency of funding disclosure was always above 95% between 2012 and 2022 (**Supplementary Table 2**).

Supplementary Table 3 shows the frequency of the PICOS categories and transparency and reproducibility practices in the protocols based in the five countries where the trials were most frequently based (the USA, UK, Iran, China, and Australia). Interventions related to nutrition education were more common in the USA (50.9%), whereas interventions related to supplementation were more common in other countries, ranging from 38.5% in China to 75.5% in Iran. Supplementation interventions were also most common in Canada, Germany, and the Netherlands. Brazil had similar frequencies of supplementation interventions and nutrition education interventions (**Supplementary Figure 4).**

Different countries used different comparator types. Other interventions were more common in the USA (38.2%), usual care was more common in Australia (36.5%), and placebo was more common in the other countries. Placebo ranged from 35.4% in China to 73.8% in Iran.

Protocols in all countries focused on adults and the elderly (23.0% [USA] to 40.3% [UK]) with a specific clinical condition (61.8% [USA] to 89.2% [Iran]).

Primary outcome choice varied with trial country. 37.6% of protocols set in China used nutritional status outcomes, 22.2% of protocols set in the UK used withdrawal or adherence outcomes, and protocols set in other countries used clinical status outcomes, from 24.1% of protocols in Australia to 75.4% of protocols in Iran.

Although still high, protocols in the USA had the lowest frequency of reporting registration information (83%) and conflicts of interest (66.4%). These transparency practices were both

seen in 98.5% of protocols of trials set in Iran. Iran had the highest proportion of mentions of SPIRIT (55.4%), the UK of TIDiER (8.3%), and Australia of CONSORT (40.9%).

Supplementary Table 4 shows the characteristics of protocols focusing on participants with cancer or cardiovascular disease. Both subgroups involved mainly adults and elderly participants and most frequently used supplementation interventions. Primary outcomes related to clinical status were more frequent in protocols involving patients with cardiovascular disease (43.3%), whereas the incidence or severity of disease was more common in protocols involving patients with cancer (25.0%). Unfunded RCTs comprised 10.5% and 1.1% of protocols involving participants with cancer and cardiovascular disease, respectively. The two subgroups had similar frequencies of reporting conflicts of interest (~14%). Only 2.2% of protocols involving participants with cardiovascular disease and 9.2% of protocols involving participants with cancer did not report study registration information.

Supplementary Discussion

A steady increase in the number of records retrieved with our search strategies for both nutrition RCTs and nutrition RCT protocols was observed in the period, as can be seen in the supplementary table 5. The proportion of records retrieved with the search strategy for nutrition RCT protocols in relation to the number of records retrieved with the search strategy for nutrition RCTs remained similar, with minimal fluctuation, across the years searched. While we cannot be sure about the raw numbers for nutrition RCTs, we have no reason to believe that the sensitivity and specificity of our two search strategies would be different (in particular because it uses a validated search strategy for nutrition RCTs (1). Thus, we believe it is safe to say that publishing RCT protocols is not a practice in decline, and we assume that the slight decrease in the proportion observed in 2020 and 2021 is due to the COVID-19 pandemic, when focus was shifted towards getting trial results published as quickly as possible.

Supplementary Table 5. Literature search of RCT protocols and RCTs on nutrition and diet-related interventions indexed in Pubmed between 2012 and 2022.

Year	Records in RCT search	Records in RCT protocol search	Proportion
2012	12,887	2,481	19.3%
2013	14,309	2,822	19.7%
2014	14,819	2,803	18.9%
2015	15,313	3,061	19.9%
2016	15,596	3,151	20.2%
2017	15,777	3,224	20.4%
2018	16,178	3,284	20.3%
2019	17,326	3,408	19.7%
2020	18,938	3,467	18.3%
2021	19,637	3,062	15.6%

References:

(1) Durão S, Kredo T, Volmink J. Validation of a search strategy to identify nutrition trials in PubMed using the relative recall method. J Clin Epidemiol. 2015;68(6):610-6. doi: 10.1016/j.jclinepi.2015.02.005.

Supplementary Box 1. Search Strategy

PubMed

Database and platform: PubMed 1946 to present (via https://pubmed.ncbi.nlm.nih.gov/)

Search filter: Cochrane Highly Sensitive Search Strategy for identifying randomised trials in MEDLINE: sensitivity- and precision-maximizing version (2008 revision); PubMed format (https://handbook-5-1.cochrane.org/chapter_6/box_6_4_b_cochrane_hsss_2008_sensprec_pubmed.htm)

#1

("Randomized controlled trial" [pt] OR "controlled clinical trial" [pt] OR randomized [tiab] OR randomised [tiab] OR placebo [tiab] OR clinical trial as topic [mesh:noexp] OR randomly [tiab] OR trial [ti]) NOT (animals [mh] NOT humans [mh])

#2

(Nutritional Sciences[mh] OR Nutritional Physiological Phenomena[mh] OR Nutrition Assessment[mh] OR Nutrition Therapy[mh] OR Nutritional and Metabolic Diseases[mh] OR nutrition*[tiab] OR diet[tiab] OR feeding[tiab] OR dietary[tiab] OR breastfeed*[tiab] OR breast feed*[tiab] OR lactation[tiab] OR bottle feed*[tiab] OR complementary feeding[tiab] OR weaning[tiab] OR enteral[tiab] OR parenteral[tiab] OR Feeding Methods[mh] OR nutritional status[tiab] OR overweight[tiab] OR obese[tiab] OR obesity[tiab] OR overnutrition[tiab] OR over nutrition[tiab] OR undernourished[tiab] OR overnourished[tiab] OR wasted[tiab] OR wasting[tiab] OR stunting[tiab] OR stunted[tiab] OR underweight[tiab] OR undernutrition[tiab] OR under nutrition[tiab] OR body weight[tiab] OR anthropometry[tiab] OR Body Weights and Measures[mh] OR growth monitoring[tiab] OR food[tiab] OR food labelling[mh] OR food assistance[mh] OR supplementary feeding[tiab] OR diet therapy[mh] OR food and beverages[mh] OR vegetable*[tiab] OR fruit*[tiab] OR meat[tiab] OR dairy[tiab] OR dietary fat*[tiab] OR starch*[tiab] OR cereal[tiab] OR food-drug interactions[mh] OR food supply[mh] OR feeding behavio*[tiab] OR eating behavio*[tiab] OR food pattern*[tiab] OR food hypersensitivity[mh] OR food deprivation[mh] OR food, organic [mh] OR micronutrient*[tiab] OR vitamin*[tiab] OR thiamin[tiab] OR riboflavin[tiab] OR niacin[tiab] OR pantothenic acid[tiab] OR pyridoxine[tiab] OR pyridoxal[tiab] OR pyridoxamine[tiab] OR biotin[tiab] OR folic acid[tiab] OR folate[tiab]OR cyanocobalamin[tiab] OR choline[tiab] OR retinol[tiab] OR ascorbic acid[tiab] OR tocopherol[tiab] OR carotenoids[tiab] OR carotene[tiab] OR cryptoxanthin[tiab] OR lutein[tiab] OR lycopene[tiab] OR zeaxanthin[tiab] OR minerals[tiab] OR calcium[tiab] OR chloride[tiab] OR magnesium[tiab] OR phosphorus[tiab] OR potassium[tiab] OR sodium[tiab] OR sulphur[tiab] OR trace element*[tiab] OR boron[tiab] OR cobalt[tiab] OR chromium[tiab] OR copper[tiab] OR fluoride[tiab] OR iodine[tiab] OR iron[tiab] OR manganese[tiab] OR molybdenum[tiab] OR selenium[tiab] OR zinc[tiab] OR trace metal*[tiab] OR macronutrient*[tiab] OR carbohydrate*[tiab] OR dietary protein*[tiab] OR saturated fat*[tiab] OR unsaturated fat*[tiab] OR mono unsaturated fat*[tiab] OR monounsaturated fat*[tiab] OR poly unsaturated fat*[tiab] OR polyunsaturated fat*[tiab] OR trans fat*[tiab] OR dietary fibre[tiab] OR dietary fiber[tiab] OR dietary salt[tiab] OR table salt[tiab] OR soft drink[tiab] OR fruit juice[tiab] OR vegetable juice[tiab] OR milk[tiab] OR tea[tiab] OR coffee[tiab] OR energy drink*[tiab] OR carbonated beverage*[tiab] OR carbonated drink*[tiab] OR prebiotics[tiab] OR probiotics[tiab] OR glycemic load[tiab] OR glycemic index[tiab] OR calories[tiab] OR kilocalories[tiab] OR kilojoules[tiab] OR caloric intake[tiab] OR energy intake[tiab]

#3
(protocol*[ti] OR study design [ti] OR trial design[ti] OR research design[ti] OR “design and methods”[ti] OR "design and rationale"[ti] OR "rationale and design"[ti] OR Research Design[mh] OR clinical protocols[mh] OR clinical trial protocol[pt])
#4
#1 AND #2 AND #3
#4 *Filters applied: From 2012/1/1 to 2022/3/24.*

Embase

Database and platform: Embase 1947 to present (via Elsevier)
Search filter: SIGN RCT filter
https://www.sign.ac.uk/what-we-do/methodology/search-filters/

#7
#6 AND (2012:py OR 2013:py OR 2014:py OR 2015:py OR 2016:py OR 2017:py OR 2018:py OR 2019:py OR 2020:py OR 2021:py OR 2022:py)
#6
#1 AND #4 AND #5
#5
“Methodology”/de OR “Clinical Protocol”/exp OR protocol:ti OR 'study design':ti OR "design and methods":ti OR "design and rationale":ti OR "rationale and design":ti
#4
#2 NOT #3
#3
'case study'/de OR 'case report':ab,ti OR 'abstract report'/de OR letter/de OR 'conference paper':it OR 'conference abstract':it OR editorial:it OR letter:it OR note:it
#2
'clinical trial'/de OR 'randomized controlled trial'/de OR 'controlled clinical trial'/de OR 'multicenter study'/de OR 'phase 3 clinical trial'/de OR ‘phase 4 clinical trial’/de OR randomization/exp OR 'single blind procedure'/de OR 'double blind procedure'/de OR 'crossover procedure'/de OR placebo/de OR 'randomi?ed controlled trial*$':ab,ti OR rct:ab,ti OR 'random* NEAR/2 allocat*':ab,ti OR 'single blind*':ab,ti OR 'double blind*':ab,ti OR ((treble:ab,ti OR triple:ab,ti) NEAR blind*:ab,ti) OR placebo*:ab,ti OR 'prospective study'/de
#1
‘Nutritional Science’/exp OR 'nutritional science':kw OR 'nutritional physiological phenomena':ab,ti OR ‘Nutrition’/exp OR 'nutritional assessment':kw OR 'nutritional support':kw OR 'diet therapy':kw OR ‘Diet Therapy’/exp OR (nutritional:kw AND 'metabolic disorder':kw) OR ‘Nutritional Disorder’/exp OR ‘Metabolic Disorder’/exp OR nutrition:ab,ti OR diet:ab,ti,kw OR feeding:ab,ti OR ‘Food’/exp OR 'dietary intake':ab,ti OR ‘Diet Restriction’/exp OR breastfeeding:ab,ti OR lactation:ab,ti OR 'bottle feeding':ab,ti OR 'complementary feeding':ab,ti OR weaning:ab,ti OR 'enteric feeding':ab,ti OR parenteral:ab,ti OR 'food intake':ab,ti OR 'nutritional status':ab,ti OR ‘Failure to Thrive’/de OR ‘Body Weight’/exp OR obesity:ab,ti OR 'obese patient':ab,ti OR overnutrition:ab,ti OR 'wasting syndrome':ab,ti OR stunting:ab,ti OR 'stunting syndrome':ab,ti OR malnutrition:ab,ti OR ‘malnutrition’/de OR 'body weight':ab,ti OR anthropometry:kw OR ('growth, development':ab,ti AND aging:ab,ti) OR food:ab,ti OR 'food packaging':kw OR ‘Food Packaging’/de OR 'food assistance':kw OR ‘Food Assistance’/de OR 'supplementary feeding':ab,ti OR ‘Food Insecurity’/exp OR vegetable:ab,ti OR fruit:ab,ti OR meat:ab,ti OR dairy:ab,ti OR 'fat intake':ab,ti OR starch:ab,ti OR cereal:ab,ti OR 'food drug interaction':ab,ti OR 'catering service':kw OR 'feeding

behavior':ab,ti OR 'eating habit':ab,ti OR 'food allergy':kw OR 'food deprivation':kw OR ‘Food Deprivation’/exp OR 'organic food':ab,ti OR ‘Organic Food’/de OR 'micronutrient intake':ab,ti OR vitamin:ab,ti OR thiamine:ab,ti OR riboflavin:ab,ti OR 'nicotinic acid':ab,ti OR 'pantothenic acid':ab,ti OR pyridoxine:ab,ti OR pyridoxal:ab,ti OR pyridoxamine:ab,ti OR biotin:ab,ti OR 'folic acid':ab,ti OR cyanocobalamin:ab,ti OR choline:ab,ti OR retinol:ab,ti OR 'ascorbic acid':ab,ti OR tocopherol:ab,ti OR carotenoid:ab,ti OR carotene:ab,ti OR cryptoxanthin:ab,ti OR xanthophyll:ab,ti OR lycopene:ab,ti OR zeaxanthin:ab,ti OR mineral:ab,ti OR calcium:ab,ti OR chloride:ab,ti OR magnesium:ab,ti OR phosphorus:ab,ti OR potassium:ab,ti OR sodium:ab,ti OR sulfur:ab,ti OR 'trace element':ab,ti OR boron:ab,ti OR cobalt:ab,ti OR chromium:ab,ti OR copper:ab,ti OR fluoride:ab,ti OR iodine:ab,ti OR iron:ab,ti OR manganese:ab,ti OR molybdenum:ab,ti OR selenium:ab,ti OR zinc:ab,ti OR 'trace metal':ab,ti OR macronutrient:ab,ti OR carbohydrate:ab,ti OR 'protein intake':ab,ti OR 'saturated fatty acid':ab,ti OR 'unsaturated fatty acid':ab,ti OR 'monounsaturated fatty acid':ab,ti OR 'polyunsaturated fatty acid':ab,ti OR 'trans fatty acid':ab,ti OR 'dietary fiber':ab,ti OR 'salt intake':ab,ti OR 'soft drink':ab,ti OR 'fruit juice':ab,ti OR 'vegetable juice':ab,ti OR milk:ab,ti OR tea:ab,ti OR coffee:ab,ti OR 'energy drink':ab,ti OR 'carbonated beverage':ab,ti OR 'prebiotic agent':ab,ti OR 'probiotic agent':ab,ti OR 'glycemic index':ab,ti OR 'glycemic load':ab,ti OR calorie:ab,ti OR 'caloric intake':ab,ti

Web of Science

Database and platform: Web of Science (All databases) 1945 to present (via Clarivate)

Search filter: Cochrane ENT group RCT filter

https://ent.cochrane.org/sites/ent.cochrane.org/files/public/uploads/rct_filters.pdf

#1

((((((TS=(protocol*)) OR TS=("study design")) OR TS=("trial design")) OR TS=("research design")) OR TS=("design and methods")) OR TS=("design and rationale")) OR TS=("rationale and design"))

#2

TS=(randomised OR randomized OR randomisation OR randomisation OR placebo* OR (random*))

#3

(((((((((((((((((((((((((((((((((((((((AK=("nutritional sciences")) OR AK=("nutritional physiological phenomena")) OR AK=("nutrition assessment")) OR AK=("nutritional support")) OR AK=(“nutrition”)) OR AK=("nutrition therapy")) OR AK=("nutritional and metabolic diseases")) OR TS=("nutrition*")) OR KP=(“nutrition”)) OR KP=(“diet”)) OR TS =("diet")) OR TS =("feeding")) OR TS =("dietary")) OR TS =("breastfeed*")) OR TS =("breast feed*")) OR TS =("lactation")) OR TS =("bottle feed*")) OR TS =("complementary feeding")) OR TS =("weaning")) OR TS =("enteral")) OR TS =("parenteral")) OR AK=("feeding methods")) OR TS =("nutritional status")) OR KP=(“nutrition support”)) OR TS =("overweight")) OR TS =("obese")) OR TS =("obesity")) OR TS =("overnutrition")) OR TS =("over nutrition")) OR TS =("undernourished")) OR TS =("overnourished")) OR TS =("wasted")) OR TS =("wasting")) OR TS =("stunting")) OR TS =("stunted")) OR TS =("underweight")) OR TS =("undernutrition")) OR TS =("under nutrition")) OR TS =("body weight")) OR TS =("anthropometry")) OR AK=("body weights and measures")) OR TS =("growth monitoring")) OR TS =("food")) OR AK=("food labeling")) OR AK=("food assistance")) OR TS =("supplementary feeding")) OR AK=("diet therapy")) OR AK=("food and beverages")) OR TS =("vegetable*")) OR TS =("fruit*")) OR TS =("meat")) OR TS =("dairy")) OR TS =("dietary fat*")) OR AB=("starch*")) OR TS =("cereal")) OR AK=("food drug interactions")) OR AK=("food supply")) OR TS

=("feeding behavio*")) OR TS =("eating behavio*")) OR TS =("food pattern*")) OR TS =("food hypersensitivity")) OR TS =("food deprivation")) OR TS =("food, organic")) OR TS =("micronutrient*")) OR TS =("vitamin*")) OR TS =("thiamin")) OR TS =("riboflavin")) OR TS =("niacin")) OR TS =("pantothenic acid")) OR TS =("pyridoxine")) OR TS =("pyridoxal")) OR TS =("pyridoxamine")) OR TS =("biotin")) OR TS =("folic acid")) OR TS =("folate")) OR TS =("cyanocobalamin")) OR TS =("choline")) OR TS =("retinol")) OR TS =("ascorbic acid")) OR TS =("tocopherol")) OR TS =("carotenoids")) OR TS =("carotene")) OR TS =("cryptoxanthin")) OR TS =("lutein")) OR TS =("lycopene")) OR TS =("zeaxanthin")) OR TS =("minerals")) OR TS =("calcium")) OR TS =("chloride")) OR TS =("magnesium")) OR TS =("phosphorus")) OR TS =("potassium")) OR TS =("sodium")) OR TS =("sulphur")) OR TS =("trace element*")) OR TS =("boron")) OR TS =("cobalt")) OR TS =("chromium")) OR TS =("copper")) OR TS =("fluoride")) OR TS =("iodine")) OR TS =("iron")) OR TS =("manganese")) OR TS =("molybdenum")) OR TS =("selenium")) OR TS =("zinc")) OR TS =("trace metal*")) OR TS =("macronutrient*")) OR TS =("carbohydrate*")) OR TS =("dietary protein*")) OR TS =("saturated fat*")) OR TS =("unsaturated fat*")) OR TS =("mono unsaturated fat*")) OR TS =("monounsaturated fat*")) OR TS =("poly unsaturated fat*")) OR TS =("polyunsaturated fat*")) OR TS =("trans fat*")) OR TS =("dietary fibre")) OR TS =("dietary fiber")) OR TS =("dietary salt")) OR TS =("table salt")) OR TS =("soft drink")) OR TS =("fruit juice")) OR TS =("vegetable juice")) OR TS =("milk")) OR TS =("tea")) OR TS =("coffee")) OR TS =("energy drink*")) OR TS =("carbonated beverage*")) OR TS =("carbonated drink*")) OR TS =("prebiotics")) OR TS =("probiotics")) OR TS =("glycaemic load")) OR TS =("glycaemic index")) OR TS =("calories")) OR TS =("kilocalories")) OR TS =("kilojoules")) OR TS =("caloric intake")) OR TS =("energy intake")

#4

#1 AND #2 AND #3

#5

#4 Timespan: 2012-01-01 to 2022-03-24 (Publication Date)

CINAHL

Database and platform: CINAHL with full text 1981 to present (via EBSCOHost)

Search filter: SIGN CINAHL for EBSCO RCT Filter (created by Mark Clowes) https://www.sign.ac.uk/assets/search-filters-randomised-controlled-trials.docx

S1

((MH "Research Protocols") OR (MH "Research Methodology") OR (MH "Study Design")) OR PT Protocol OR (TI (protocol* OR "study design" OR "trial design" OR "research design" OR "design and methods" OR "design and rationale" OR "rationale and design"))

S2

((MH "Nutrition+") OR (MH "Nutritional Physiology+") OR (MH "Nutritional Assessment") OR (MH "Diet Therapy+") OR (MH "Nutritional and Metabolic Diseases+") OR (MH "Feeding Methods") OR (MH “Diet+”)) OR (TI (nutrition* OR diet OR feeding OR dietary OR breastfeed* OR "breast feed*" OR lactation OR "bottle feed*" OR "complementary feeding" OR weaning OR enteral OR parenteral) OR AB (nutrition* OR diet OR feeding OR dietary OR breastfeed* OR "breast feed*" OR lactation OR "bottle feed*" OR "complementary feeding" OR weaning OR enteral OR parenteral)) OR (TI ("nutritional status" OR overweight OR obese OR obesity OR overnutrition OR "over nutrition" OR undernourished OR overnourished OR wasted OR wasting OR stunting OR stunted OR underweight OR undernutrition OR "under nutrition" OR "body weight" OR anthropometry) OR AB

("nutritional status" OR overweight OR obese OR obesity OR overnutrition OR "over nutrition" OR undernourished OR overnourished OR wasted OR wasting OR stunting OR stunted OR underweight OR undernutrition OR "under nutrition" OR "body weight" OR anthropometry)) OR ((MH "Body Weights and Measures") OR (MH "Anthropometry") OR (MH "Body Weight") OR (MH "Food Labeling") OR (MH "Food Assistance") OR (MH "Food and Beverages+")) OR (TI ("growth monitoring" OR food OR "supplementary feeding" OR vegetable* OR fruit* OR meat OR dairy OR "dietary fat*" OR starch* OR cereal) OR AB ("growth monitoring" OR food OR "supplementary feeding" OR vegetable* OR fruit* OR meat OR dairy OR "dietary fat*" OR starch* OR cereal)) OR ((MH "Drug-Food Interactions") OR (MH "Food Supply") OR (MH "Organic Food") OR (MH "Food Hypersensitivity")) OR (TI ("feeding behavio*" OR "eating behavio*" OR "food pattern*" OR micronutrient* OR vitamin* OR thiamin OR riboflavin OR niacin OR "pantothenic acid" OR pyridoxine OR pyridoxal OR pyridoxamine OR biotin OR "folic acid" OR folate OR cyanocobalamin OR choline OR retinol OR "ascorbic acid" OR tocopherol OR carotenoids OR carotene OR cryptoxanthin) OR AB ("feeding behavio*" OR "eating behavio*" OR "food pattern*" OR micronutrient* OR vitamin* OR thiamin OR riboflavin OR niacin OR "pantothenic acid" OR pyridoxine OR pyridoxal OR pyridoxamine OR biotin OR "folic acid" OR folate OR cyanocobalamin OR choline OR retinol OR "ascorbic acid" OR tocopherol OR carotenoids OR carotene OR cryptoxanthin)) OR (TI (lutein OR lycopene OR zeaxanthin OR minerals OR calcium OR chloride OR magnesium OR phosphorus OR potassium OR sodium OR sulphur OR "trace element*" OR boron OR cobalt OR chromium OR copper OR fluoride OR iodine OR iron OR manganese OR molybdenum OR selenium OR zinc OR "trace metal*" OR macronutrient* OR carbohydrate* OR "dietary protein*") OR AB (lutein OR lycopene OR zeaxanthin OR minerals OR calcium OR chloride OR magnesium OR phosphorus OR potassium OR sodium OR sulphur OR "trace element*" OR boron OR cobalt OR chromium OR copper OR fluoride OR iodine OR iron OR manganese OR molybdenum OR selenium OR zinc OR "trace metal*" OR macronutrient* OR carbohydrate* OR "dietary protein*")) OR (TI ("saturated fat*" OR "unsaturated fat*" OR "mono unsaturated fat*" OR "monounsaturated fat*" OR "poly unsaturated fat*" OR "polyunsaturated fat*" OR "trans fat*" OR "dietary fibre" OR "dietary fiber" OR "dietary salt" OR "table salt" OR "soft drink" OR "fruit juice" OR "vegetable juice") OR AB ("saturated fat*" OR "unsaturated fat*" OR "mono unsaturated fat*" OR "monounsaturated fat*" OR "poly unsaturated fat*" OR "polyunsaturated fat*" OR "trans fat*" OR "dietary fibre" OR "dietary fiber" OR "dietary salt" OR "table salt" OR "soft drink" OR "fruit juice" OR "vegetable juice")) OR (TI (milk OR tea OR coffee OR "energy drink*" OR "carbonated beverage*" OR "carbonated drink*" OR prebiotics OR probiotics OR "glycemic load" OR "glycemic index" OR calories OR kilocalories OR kilojoules OR "caloric intake" OR "energy intake") OR AB (milk OR tea OR coffee OR "energy drink*" OR "carbonated beverage*" OR "carbonated drink*" OR prebiotics OR probiotics OR "glycemic load" OR "glycemic index" OR calories OR kilocalories OR kilojoules OR "caloric intake" OR "energy intake"))

S3

TX allocat* random* OR (MH "Quantitative Studies") OR (MH "Placebos") OR TX placebo* OR TX random* allocat* OR (MH "Random Assignment") OR TX randomi* control* trial* OR (TX ((singl* n1 blind*) or (singl* n1 mask*)) or TX ((doubl* n1 blind*) or (doubl* n1 mask*)) or TX ((tripl* n1 blind*) or (tripl* n1 mask*)) or TX ((trebl* n1 blind*) or (trebl* n1 mask*))) OR TX clinic* n1 trial* OR PT Clinical trial OR (MH "Clinical Trials+")

S4

S1 AND S2 AND S3

S5

S4 (LIMITADORES 202203 - 201201)

Global Health

Database and platform: Global Health 1973 to 2022 Week 12 (via OVID)

Search filter: RCT filter adapted from the Cochrane Highly Sensitive Search Strategy for identifying randomised trials in MEDLINE: sensitivity- and precision-maximising version (2008 revision)

1. (protocol$ OR "study design$" OR "trial design$" OR "research design$" OR "design and methods" OR "design and rationale" OR "rationale and design").ti.

2. Methodology/ OR Experimental Design/

3. 1 OR 2

4. exp Nutrition/ OR exp Nutrition Research/ OR exp Human Feeding/ OR Diets/ OR Diet/

5. Nutrition Physiology/ OR Nutritional Assessment/ OR Nutritional Disorders/ OR Nutritional Intervention/

6. (nutrition$ OR diet OR feeding OR dietary OR breastfeed$ OR "breast feed$" OR lactation OR "bottle feed$" OR "complementary feeding" OR weaning OR enteral OR parenteral).ti,ab.

7. ("nutritional status" OR overweight OR obese OR obesity OR overnutrition OR "over nutrition" OR undernourished OR overnourished OR wasted OR wasting OR stunting OR stunted OR underweight OR undernutrition OR "under nutrition" OR "body weight" OR anthropometry).ti,ab.

8. Body Weight/ OR Nutrition Labelling/ OR Diet Treatment/ OR Food Supply/ OR Nutrient Drug Interactions/ OR Food Deprivation/

9. exp Body Measurements/ OR exp Foods/ OR exp Therapeutic Diets/ OR exp Food Allergies/

10. ("growth monitoring" OR food OR "food assistance" OR "supplementary feeding" OR vegetable$ OR fruit$ OR meat$ OR dairy$ OR "dietary fat$" OR starch$ OR cereal OR "feeding behavio$" OR "eating behavio$" OR "food pattern$").ti,ab.

11. ("food hypersensitivit$" OR "organic food" OR micronutrient$ OR vitamin$ OR thiamin OR riboflavin OR niacin OR "pantothenic acid" OR pyridoxine OR pyridoxal OR pyridoxamine OR biotin OR "folic acid" OR folate OR cyanocobalamin OR choline OR retinol OR "ascorbic acid" OR tocopherol OR carotenoids OR carotene OR cryptoxanthin OR lutein OR lycopene OR zeaxanthin OR minerals OR calcium OR chloride OR magnesium OR phosphorus OR potassium OR sodium OR sulphur OR "trace element$" OR boron OR cobalt OR chromium OR copper OR fluoride OR iodine OR iron OR manganese OR molybdenum OR selenium OR zinc OR "trace metal$").ti,ab.

12. (macronutrient$ OR carbohydrate$ OR "dietary protein$" OR "saturated fat$" OR "unsaturated fat$" OR "mono unsaturated fat$" OR "monounsaturated fat$" OR "poly unsaturated fat$" OR "polyunsaturated fat$" OR "transfat$" OR "dietary fibre" OR "dietary fiber" OR "dietary salt" OR "table salt" OR "soft drink" OR "fruit juice" OR "vegetable juice" OR milk OR tea OR coffee OR "energy drink$" OR "carbonated beverage$" OR "carbonated drink$" OR prebiotics OR probiotics OR "glycemic load" OR "glycemic index" OR calories OR kilocalories OR kilojoules OR "caloric intake" OR "energy intake").ti,ab.

13. 4 OR 5 OR 6 OR 7 OR 8 OR 9 OR 10 OR 11 OR 12

14. Randomized controlled trials/ OR Clinical Trials/ OR Placebos/

15. (randomized OR randomised OR randomly OR placebo OR trial$).ti,ab.

16. 14 OR 15

17. 3 AND 13 AND 16

18. Limit 17 to yr="2012-2022"

PsycInfo

Database and platform: PsycINFO 1806 to present (via OVID)

Search filter: Cochrane ENT Group RCT search filter for PsycInfo (OVID) https://ent.cochrane.org/sites/ent.cochrane.org/files/public/uploads/rct_filters.pdf

1. (protocol$ OR "study design$" OR "trial design$" OR "research design$" OR "design and methods" OR "design and rationale" OR "rationale and design").ti.

2. ("Research Design" OR "clinical protocols").mh.

3. Methodology/

4. Experimental Design/

5. 1 OR 2 OR 3 OR 4

6. ("Nutritional Sciences" OR "Nutritional Physiological Phenomena" OR "Nutrition Assessment" OR "Nutrition Therapy" OR "Nutritional and Metabolic Diseases" OR "Feeding Methods" OR "Body Weights and Measures" OR "Food Labelling" OR "Food Assistance" OR "Diet Therapy" OR "Food and Beverages" OR "Food-drug Interactions" OR "Food Supply" OR "Food Hypersensitivity" OR "Food Deprivation" OR "Food, Organic").mh.

7. exp Nutrition/ OR exp "Nutritional Deficiencies"/ OR exp "Metabolism Disorders"/ OR exp Diets/ OR exp Food/

8. "Eating Behavior"/ OR "Failure to Thrive"/ OR "Body Weight"/ OR "Beverages (Nonalcoholic)"/ OR "Food Insecurity"/ OR "Food Allergies"/ OR "Food Deprivation"/ OR "Food Intake"/ OR "Dietary Supplements"/ OR "Dietary Restraint"/ OR "Food Preferences"/

9. (nutrition$ OR diet OR feeding OR dietary OR breastfeed$ OR "breast feed$" OR lactation OR "bottle feed$" OR "complementary feeding" OR weaning OR enteral OR parenteral OR "nutritional status" OR overweight OR obese OR obesity OR overnutrition OR "over nutrition" OR undernourished OR overnourished OR wasted OR wasting OR stunting OR stunted OR underweight OR undernutrition OR "under nutrition" OR "body weight" OR anthropometry).ti,ab.

10. ("growth monitoring" OR food OR "supplementary feeding" OR vegetable$ OR fruit$ OR meat OR dairy OR "dietary fat$" OR starch$ OR cereal OR "feeding behavio$" OR "eating behavio$" OR "food pattern$" OR micronutrient$).ti,ab.

11. (vitamin$ OR thiamin OR riboflavin OR niacin OR pantothenic acid OR pyridoxine OR pyridoxal OR pyridoxamine OR biotin OR folic acid OR folate OR cyanocobalamin OR choline OR retinol OR ascorbic acid OR tocopherol OR carotenoids OR carotene OR cryptoxanthin OR lutein OR lycopene OR zeaxanthin OR minerals OR calcium OR chloride OR magnesium OR phosphorus OR potassium OR sodium OR sulphur OR "trace element$" OR boron OR cobalt OR chromium OR copper OR fluoride OR iodine OR iron OR manganese OR molybdenum OR selenium OR zinc OR "trace metal$").ti,ab.

12. (macronutrient$ OR carbohydrate$ OR "dietary protein$" OR "saturated fat$" OR "unsaturated fat$" OR "mono unsaturated fat$" OR "monounsaturated fat$" OR "poly unsaturated fat$" OR "polyunsaturated fat$" OR "trans fat$" OR "dietary fibre" OR "dietary fiber" OR "dietary salt" OR "table salt").ti,ab.

13. ("soft drink" OR "fruit juice" OR "vegetable juice" OR milk OR tea OR coffee OR "energy drink$" OR "carbonated beverage$" OR "carbonated drink$" OR prebiotics OR probiotics OR "glycemic load" OR "glycemic index" OR calories OR kilocalories OR kilojoules OR "caloric intake" OR "energy intake").ti,ab.

14. 6 OR 7 OR 8 OR 9 OR 10 OR 11 OR 12 OR 13

15. Placebo/

16. ((blind* or mask*) and (single or double or triple or treble)).tw.

17. (randomised or randomized or randomisation or randomization or (random* and (allocat* or assign*)) or factorial* or placebo* or crossover* or (cross adj over*)).tw.

18. 15 or 16 or 17

19. 5 AND 14 AND 18

20. limit 19 to yr="2012-2022"

Supplementary Box 2. Data extraction form

Draft Data Extraction Protocols Of Nutrition or Diet-related RCTs

Number of report	______________________
Confirmation of eligibility	○ no<br>○ yes
Reason of no eligibility:	○ it is not a protocol<br>○ intervention is not related to nutrition or diet<br>○ it is not a protocol of RCT<br>○ it is a preprint<br>○ it is an abstract<br>○ it is a duplicate
First author	______________________
Year of publication	______________________
Journal of publication	______________________
PICOTS_ population	☐ Pregnant women<br>☐ Mother and infant pairs<br>☐ Infants<br>☐ Children and preschool-aged children<br>☐ Adolescents<br>☐ Adults<br>☐ Elderly<br>☐ Adults and elderly<br>☐ Postmenopausal women<br>☐ Participants with a clinical condition (s)<br>☐ Families
PICOTS_ population_clinical condition	______________________
PICOTS_ population_cancer	○ no<br>○ yes
PICOTS_ population_CVD	○ no<br>○ yes

PICOTS_intervention_complexity	◯ Only nutrition or diet-related intervention<br>◯ Nutrition or diet-related intervention combined with exercise<br>◯ Nutrition or diet-related intervention combined with drugs<br>◯ Nutrition or diet-related intervention combined with other medical care<br>◯ Nutrition or diet-related intervention as part of a lifestyle intervention<br>◯ Nutrition or diet-related intervention as part of a health intervention
PICOTS_intervention_type	◯ Food (whole food, food products, specially formulated foods)<br>◯ Breastfeeding, complementary feeding, weaning<br>◯ Complete diet or dietary patterns<br>◯ Complete nutrition formulas (enteral or parenteral)<br>◯ Supplementation or supplements (single or multiple nutrients, bioactive non-nutrients, plant components)<br>◯ Nutrition education, counseling and coordination of care<br>◯ Other, if no component of intervention could be categorized as any of the above
PICOTS_intervention_type_details	______________________
Number of arms (groups)	◯ 2<br>◯ 3<br>◯ 4<br>◯ 5 or more
PICOTS_Control	◯ Placebo<br>◯ Usual care<br>◯ Other intervention<br>◯ No control (such as wait list)
PICOTS_duration	______________________<br>(in months)
PICOTS_primary outcome	◯ not specified<br>◯ specified

primary outcome	☐ Mortality<br>☐ Clinical status (clinical or biochemical measures)<br>☐ Nutritional status (anthropometry, body composition, nutrition diagnosis)<br>☐ Functional status<br>☐ Frequency or severity of disease<br>☐ Diet quality and/or variety<br>☐ Food/ nutrient/ dietary intake<br>☐ Diet-related behaviors<br>☐ Other non-dietary behaviors<br>☐ Withdrawal from the study, drop-out or adherence-related<br>☐ Adverse events, side-effects and/or safety<br>☐ Cost-effectiveness or economic<br>☐ Quality of life<br>☐ Breastfeeding<br>☐ Other
other primary outcome description	__________________________
Study Design	○ Parallel<br>○ Crossover
Pilot study	○ No<br>○ Yes
Study Design_1	○ Cluster RCT<br>○ No cluster RCT
Study Design_2	○ Factorial RCT<br>○ No factorial RCT
Study Design_Framework	○ Superiority<br>○ Non - inferiority<br>○ Equivalence<br>○ Exploratory<br>○ Not reported
Study local	○ unicentric<br>○ multicentric
Study country	__________________________
Registered protocol	○ no<br>○ yes
Reference to SPIRIT	○ no<br>○ yes
Reference to CONSORT	○ no<br>○ yes
Reference to TIDieR	○ no<br>○ yes

Reference to CONSERVE	◯ no<br>◯ yes
Funding reporting	◯ no funding<br>◯ funding reported<br>◯ none statement about funding
COI reporting	◯ no COI<br>◯ COI reported<br>◯ none statement on COI

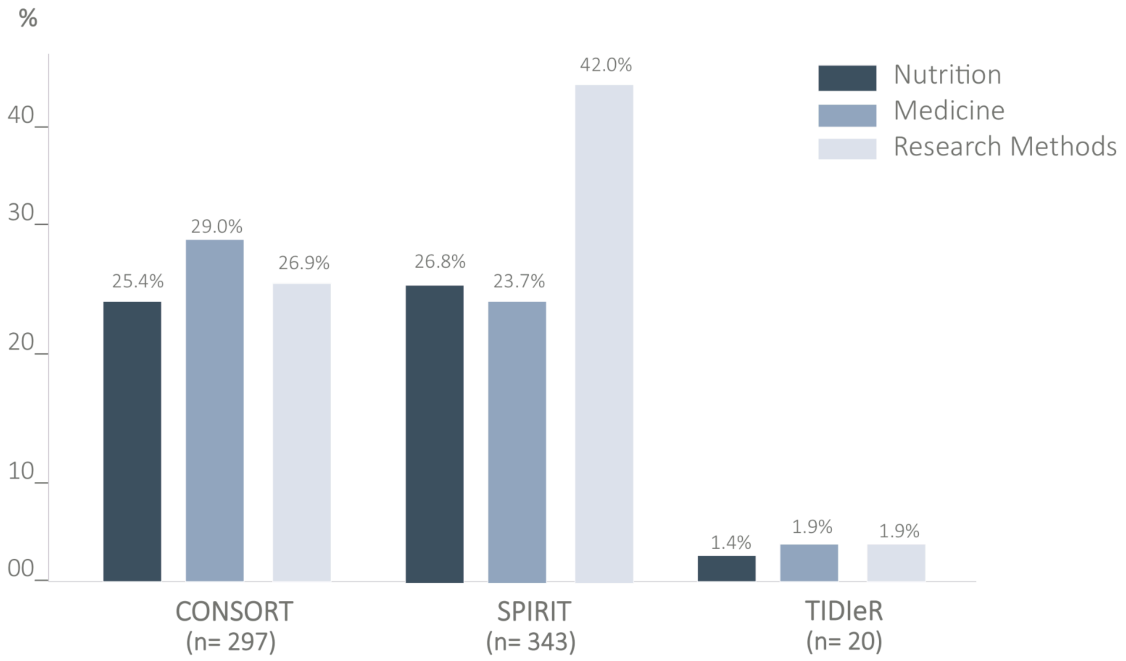

Supplementary Figure 1. Relative frequency of nutrition- and diet-related RCT protocols published 2012-2022 that referenced the CONSORT, SPIRIT, and TIDieR reporting guidelines, grouped by type of journal.

2A

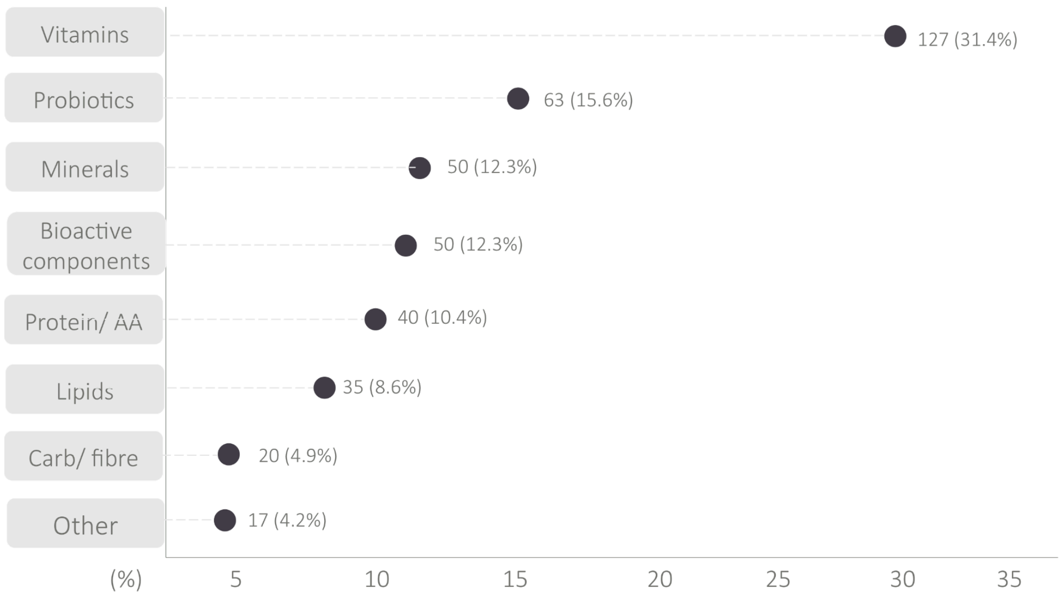

2B

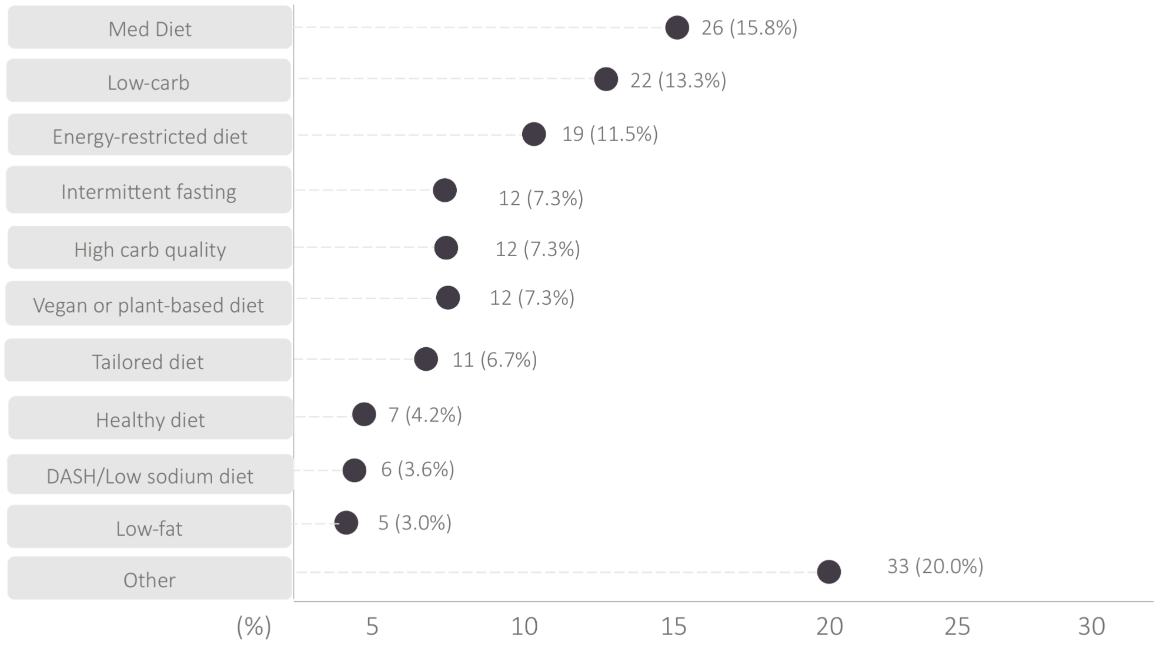

Supplementary Figure 2: Absolute and relative frequencies of the supplements (A; n = 405) and complete diet or dietary patterns (B; n = 165) used as interventions in protocols for nutrition- and diet-related RCTS published between 2012 and 2022.

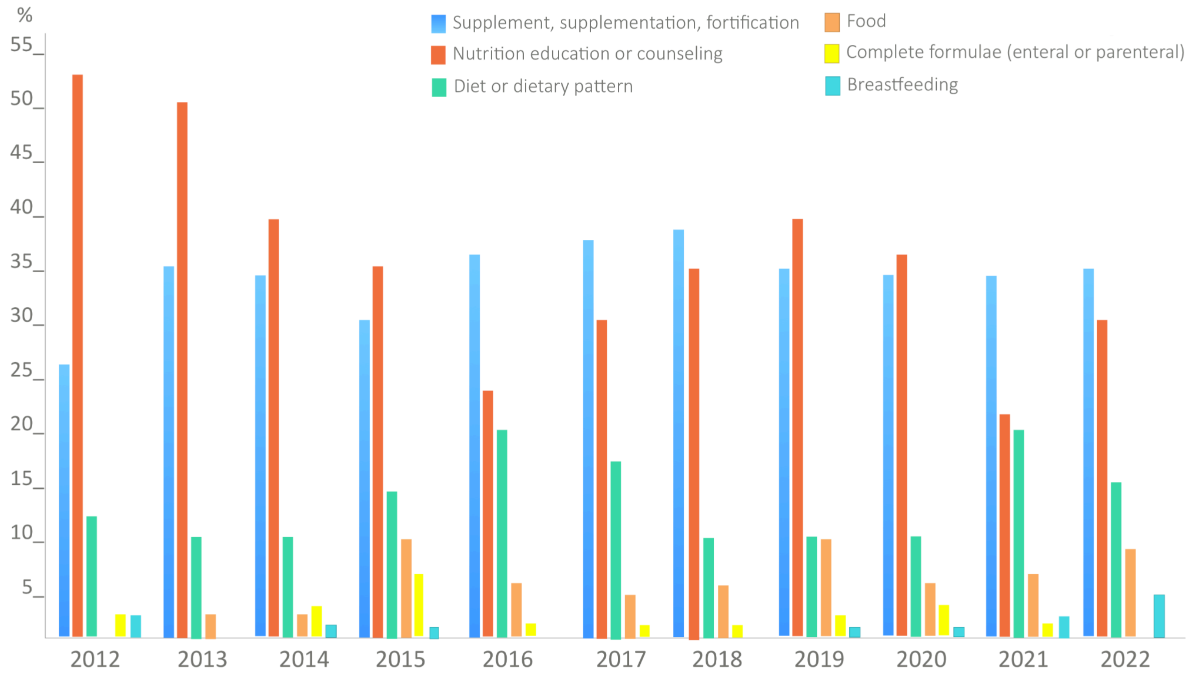

Supplementary Figure 3: Frequency of intervention categories used in protocols of nutrition- and diet-related RCTs published 2012-2022, by publication year.

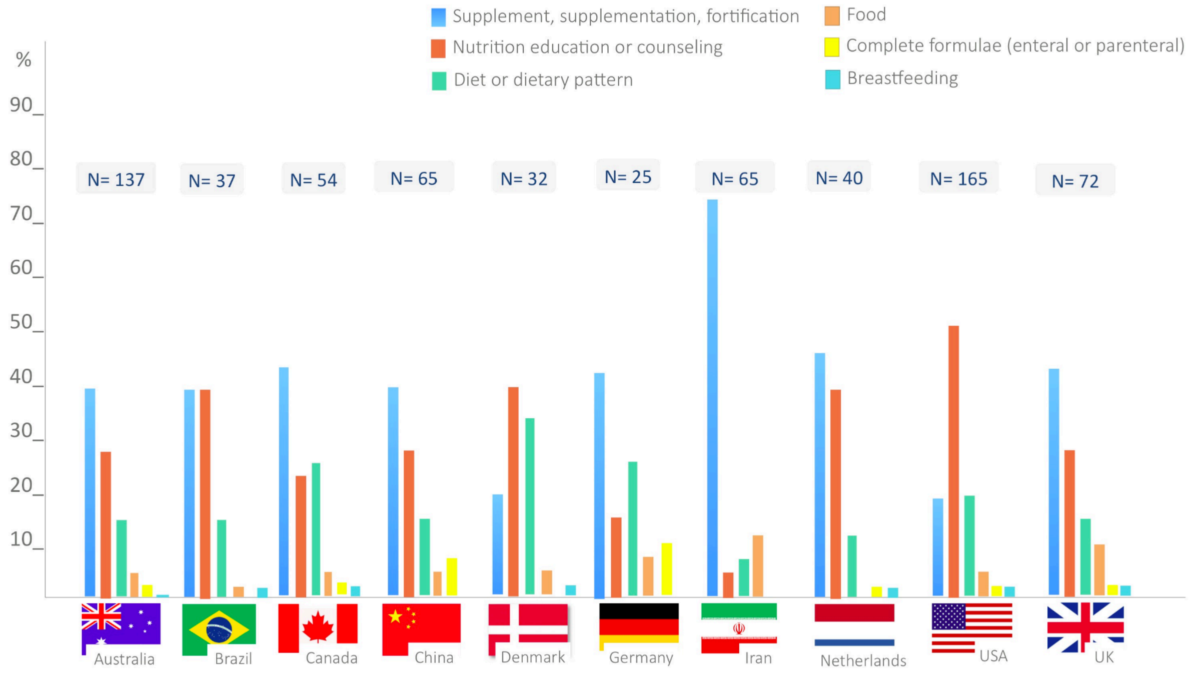

Supplementary Figure 4: Frequency of intervention categories used in protocols of nutrition- and diet-related RCTs published 2012-2022, by country where the trial is set, showing countries with at least 25 protocols.

Supplementary Table 1. Proportion of protocols of nutrition- and diet-related RCTs published 2012-2022 that mention the CONSORT, SPIRIT, and TIDieR reporting guidelines, by whether the journal the protocol is published in endorses that reporting guideline.

	**Endorsement by the journal that published the protocol**					
	**SPIRIT**		**CONSORT**		**TIDIeR**	
**Mentioned by the protocol**	Yes	No	Yes	No	Yes	No
Yes	123 (25.4%)	220 (37.7%)	292 (28.7%)	5 (9.6%)	0 (0%)	20 (1.9%)
No	362 (74.6%)	363 (62.3%)	724 (71.3%)	47 (90.4%)	7 (100%)	1041 (98.1%)
Total	485 (45.4%)	583 (54.6%)	1016 (95.1%)	52 (4.9%)	7 (0.7%)	1061 (99.3%)

Supplementary Table 2. Absolute and relative frequencies of PICOS categories and transparency and reproducibility principles used in protocols of nutrition- and diet-related RCT trials published 2012-2022, stratified by the year of publication (n = 1068)

		**2012**	**2013**	**2014**	**2015**	**2016**	**2017**	**2018**	**2019**	**2020**	**2021**	**2022**
	N	32	71	76	79	86	102	107	156	163	155	41
P - Population (age-stage)												
Infant	66 (6.2%)	2 (6.2%)	2 (2.8%)	5 (6.6%)	8 (10.1%)	5 (5.8%)	8 (7.8%)	5 (4.7%)	8 (5.1%)	8 (4.9%)	9 (5.8%)	6 (14.6%)
Children	132 (12.4%)	6 (18.8%)	14 (19.7%)	7 (9.2%)	11 (13.9%)	10 (11.6%)	13 (12.7%)	14 (13.1%)	26 (16.7%)	16 (9.8%)	15 (9.7%)	0 (0%)
Adolescent	63 (5.9%)	2 (6.2%)	5 (7.0%)	1 (1.3%)	9 (11.4%)	6 (7.0%)	9 (8.8%)	5 (4.7%)	9 (5.8%)	10 (6.1%)	6 (3.9%)	1 (2.4%)
Adult	251 (23.5%)	7 (21.9%)	16 (22.5%)	15 (19.7%)	21 (26.6%)	20 (23.3%)	30 (29.4%)	22 (20.6%)	40 (25.6%)	40 (24.5%)	32 (20.6%)	8 (19.5%)
Adult and elderly	350 (32.8%)	5 (15.6%)	22 (31.0%)	30 (39.5%)	22 (27.8%)	26 (30.2%)	30 (29.4%)	37 (34.6%)	48 (30.8%)	56 (34.4%)	61 (39.4%)	13 (31.7%)
Elderly	76 (7.1%)	4 (12.5%)	4 (5.6%)	5 (6.6%)	4 (5.1%)	4 (4.7%)	2 (2.%)	11 (10.3%)	11 (7.1%)	14 (8.6%)	14 (9.0%)	3 (7.3%)

		2012	2013	2014	2015	2016	2017	2018	2019	2020	2021	2022
Pregnant	99 (9.3%)	3 (9.4%)	9 (12.7%)	10 (13.2%)	5 (6.3%)	9 (10.5%)	11 (10.8%)	11 (10.3%)	9 (5.8%)	13 (8.0%)	12 (7.7%)	7 (17.1%)
Postmenopausal women	9 (0.8%)	0 (0%)	0 (0%)	1 (1.3%)	0 (0%)	4 (4.7%)	0 (0%)	0 (0%)	0 (0%)	1 (0.6%)	3 (1.9%)	0 (0%)
Families	53 (5.0%)	3 (9.4%)	5 (7.0%)	1 (1.3%)	6 (7.6%)	4 (4.7%)	4 (3.9%)	4 (3.7%)	8 (5.1%)	12 (7.4%)	5 (3.2%)	1 (2.4%)
Mother and infant	19 (1.8%)	0 (0%)	2 (2.8%)	2 (2.6%)	2 (1.5%)	1 (1.2%)	1 (1.0%)	3 (2.8%)	2 (1.3%)	2 (1.2%)	3 (1.9%)	1 (2.4%)
P - population (clinical condition)												
No specific disease	392	16 (50.0%)	27 (38.0%)	27 (35.5)	26 (32.9%)	34 (39.5%)	43 (42.2%)	38 (35.5%)	67 (42.9%)	42 (25.8%)	59 (38.1%)	13 (31.7%)
With a specific disease	676 (63.3%)	16 (50.0%)	44 (62.0%)	49 (64.5)	53 (67.1%)	52 (60.5%)	59 (57.8%)	69 (64.5%)	89 (57.1%)	121 (74.2%)	96 (61.9%)	28 (68.3%)
I - Intervention (type)												
Isolated	724 (67.8%)	18 (56.2%)	34 (47.9%)	52 (68.4%)	47 (59.5%)	61 (70.9%)	69 (67.6%)	70 (65.4%)	106 (67.9%)	114 (69.9%)	119 (76.8%)	34 (82.9%)
Combined with exercise	98 (9.2%)	3 (9.4%)	7 (9.9%)	7 (9.2%)	10 (12.7%)	8 (9.3%)	11 (10.8%)	14 (13.1%)	12 (7.7%)	19 (11.7%)	7 (4.5%)	0 (0%)

		2012	2013	2014	2015	2016	2017	2018	2019	2020	2021	2022
Combined with drug	18 (1.7%)	0 (0%)	1 (1.4%)	0 (0%)	1 (1.3%)	0 (0%)	2 (2.0%)	3 (2.8%)	4 (2.6%)	4 (2.5%)	2 (1.3%)	1 (2.4%)
Combined with other medical care	18 (1.7%)	0 (0%)	1 (1.4%)	0 (0%)	2 (2.5%)	2 (2.3%)	0 (0%)	3 (2.8%)	3 (1.9%)	3 (1.8%)	4 (2.6%)	0 (0%)
Part of lifestyle modification	127 (11.9%)	7 (21.9%)	20 (28.2%)	10 (13.2%)	15 (19.0%)	6 (7.0%)	14 (13.7%)	8 (7.5%)	15 (9.6%)	14 (8.6%)	14 (9.0%)	4 (9.8%)
Part of health programme	83 (7.8%)	4 (12.5%)	8 (11.3%)	7 (9.2%)	4 (5.1%)	9 (10.5%)	6 (5.9%)	9 (8.4%)	16 (10.3%)	9 (5.5%)	9 (5.8%)	2 (4.9%)
I - Intervention (category)												
Food	78 (7.3%)	0 (0%)	2 (2.8%)	3 (3.9%)	7 (8.9%)	6 (7.0%)	6 (5.9%)	8 (7.5%)	17 (10.9%)	12 (7.4%)	13 (8.4%)	4 (9.8%)
Diet or dietary pattern	165 (15.4%)	4 (12.5%)	7 (9.9%)	8 (10.5%)	12 (15.2%)	20 (23.3%)	19 (18.6%)	14 (13.1%)	17 (10.9%)	24 (14.7%)	33 (21.3%)	7 (17.1%)
Supplementation	405 (37.9%)	9 (28.1%)	26 (36.6%)	26 (34.2%)	24 (30.4%)	33 (38.4%)	40 (39.2%)	44 (41.1%)	56 (35.9%)	69 (42.3%)	63 (40.6%)	15 (36.6%)
Complete formulae	37 (3.5%)	1 (3.1%)	0 (0%)	4 (5.3%)	6 (7.6%)	2 (2.3%)	3 (2.9%)	2 (1.9%)	6 (3.8%)	9 (5.5%)	4 (2.6%)	0 (0%)
Nutrition education	354 (33.1%)	17 (53.1%)	36 (50.7%)	31 (40.8%)	28 (35.4%)	21 (24.4%)	32 (31.4%)	39 (36.4%)	57 (36.5%)	46 (28.2%)	35 (22.6%)	12 (29.3%)

		2012	2013	2014	2015	2016	2017	2018	2019	2020	2021	2022
Breastfeeding	17 (1.6%)	1 (3.1%)	0 (0%)	2 (2.6%)	1 (1.3%)	0 (0%)	0 (0%)	0 (0%)	2 (1.3%)	2 (1.2%)	6 (3.9%)	3 (7.3%)
Other	12 (1.1%)	0 (0%)	0 (0%)	2 (2.6%)	1 (1.3%)	4 (4.7%)	2 (2.0%)	0 (0%)	1 (0.6%)	1 (0.6%)	1 (0.6%)	0 (0%)
C - Comparator												
Placebo	362 (33.9%)	7 (21.9%)	26 (36.6%)	23 (30.3%)	26 (32.9%)	29 (33.7%)	36 (35.3%)	30 (28.0%)	55 (35.3%)	58 (35.6%)	58 (37.4%)	14 (34.1%)
Other intervention	289 (27.1%)	12 (37.5%)	14 (19.7%)	20 (26.3%)	23 (29.1%)	24 (27.9%)	31 (30.4%)	32 (29.9%)	36 (23.1%)	48 (29.4%)	40 (25.8%)	9 (22.0%)
Usual care	316 (29.6%)	7 (21.9%)	22 (31.0%)	25 (32.9%)	27 (34.2%)	25 (29.1%)	27 (26.5%)	35 (32.7%)	48 (30.8%)	38 (23.3%)	46 (29.7%)	16 (39.0%)
No control	101 (9.5%)	6 (18.8%)	9 (12.7%)	8 (10.5%)	3 (3.8%)	8 (9.3%)	8 (7.5%)	10 (9.3%)	17 (10.9%)	19 (11.7%)	11 (7.1%)	2 (4.9%)
O - Primary outcome (three most frequent categories)												
Clinical status	308 (28.8%)	6 (18.8%)	22 (31.0%)	21 (27.6%)	19 (24.1%)	27 (31.4%)	32 (31.4%)	22 (20.6%)	45 (28.8%)	45 (27.6%)	58 (37.4%)	11 (26.8%)
Nutritional status	247 (23.1%)	15 (46.9%)	25 (35.2%)	12 (15.8%)	18 (22.8%)	23 (26.7%)	23 (22.5%)	23 (21.5%)	36 (23.1%)	36 (22.1%)	27 (17.4%)	9 (22.0%)

		2012	2013	2014	2015	2016	2017	2018	2019	2020	2021	2022
Frequency or severity of disease	238 (22.3%)	5 (15.6%)	17 (23.9%)	17 (22.4%)	22 (27.8%)	18 (20.9%)	21 (20.6%)	20 (18.7%)	31 (19.9%)	42 (25.8%)	35 (22.6%)	10 (24.4%)
S - Study design												
Crossover	54	1 (3.1%)	4 (5.6%)	5 (6.6%)	4 (5.1%)	5 (5.8%)	6 (5.9%)	6 (5.6%)	7 (4.5%)	7 (4.3%)	7 (4.5%)	2 (4.9%)
Parallel	1014	31 (96.9%)	67 (94.4%)	71 (93.4%)	75 (94.9%)	81 (94.2%)	96 (94.1%)	101 (94.4%)	149 (95.5%)	156 (95.7%)	148 (95.5%)	39 (95.5%)
Practices of transparency and reproducibility												
Registration of protocol	1006 (94.2%)	30 (93.8%)	61 (85.9%)	72 (94.7%)	72 (91.1%)	80 (93.0%)	100 (98.0%)	102 (95.3%)	147 (94.2%)	156 (95.7%)	147 (94.8%)	39 (95.1%)
Conflicts of interest statement	953 (89.2%)	29 (90.6%)	58 (81.7%)	69 (90.8%)	71 (89.9%)	73 (84.9%)	96 (94.1%)	95 (88.8%)	136 (87.2%)	146 (89.6%)	141 (91.0%)	39 (95.1%)
Funding statement	1043 (97.7%)	32 (100%)	68 (95.8%)	73 (96.1%)	76 (96.2%)	82 (95.3%)	100 (98.0%)	105 (98.1%)	154 (98.7%)	160 (98.2%)	152 (98.1%)	41 (100%)

Supplementary Table 3. Absolute and relative frequency of PICOS categories and transparency and reproducibility principles used in protocols of nutrition- and diet-related RCT trials published 2012-2022 that are based in the five most common countries, stratified by country.

**Characteristics**	**USA (n = 165)**	**UK (n = 72)**	**Iran (n = 65)**	**China (n = 65)**	**Australia (n = 137)**
P - population (life-stage)					
Infant	6 (3.6%)	4 (5.6%)	0 (0%)	4 (6.2%)	4 (2.9%)
Children	32 (19.4%)	4 (5.6%)	2 (3.1%)	6 (9.2%)	16 (11.7%)
Adolescent	11 (6.7%)	4 (5.6%)	3 (4.6%)	1 (1.5%)	7 (5.1%)
Adult	37 (22.4%)	20 (27.8%)	31 (47.7%)	14 (21.5%)	35 (25.5%)
Adult and elderly	38 (23.0%)	29 (40.3%)	26 (40.0%)	24 (36.9%)	47 (34.3%)
Elderly	6 (3.6%)	8 (11.1%)	0 (0%)	3 (4.6%)	12 (8.8%)
Pregnant	16 (9.7%)	2 (2.8%)	2 (3.1%)	6 (9.2%)	14 (10.2%)
Postmenopausal women	3 (1.8%)	0 (0%)	1 (1.5%)	3 (4.6%)	1 (0.7%)
Families	33 (20.0%)	1 (1.4%)	0 (0%)	4 (6.2%)	2 (1.5%)
Mother and infant	3 (1.8%)	1 (1.4%)	0 (0%)	1 (1.5%)	0 (0%)
P - population (clinical condition)					
No disease	63 (38.2%)	22 (30.6%)	7 (10.8%)	18 (27.7%)	48 (35.0%)
Specific disease	102 (61.8%)	50 (69.4%)	58 (89.2%)	47 (72.3%)	89 (65.0%)

**Characteristics**	**USA (n = 165)**	**UK (n = 72)**	**Iran (n = 65)**	**China (n = 65)**	**Australia (n = 137)**
I - Intervention (type of intervention)					
Isolated	89 (53.9%)	48 (66.7%)	61 (93.8%)	48 (73.8%)	95 (69.3%)
Combined with exercise	19 (11.5%)	9 (12.5%)	1 (1.5%)	8 (12.3%)	14 (10.2%)
Combined with drug	0 (0%)	4 (5.6%)	0 (0%)	3 (4.6%)	0 (0%)
Combined with other medical care	1 (0.6%)	1 (1.4%)	1 (1.5%)	0 (0%)	2 (1.5%)
Part of lifestyle modification	34 (20.6%)	7 (9.7%)	1 (1.5%)	2 (3.1%)	18 (13.1%)
Part of health programme	22 (13.3%)	3 (4.2%)	1 (1.5%)	4 (6.2%)	8 (5.8%)
I - Intervention (categories of intervention)					
Food	10 (6.1%)	8 (11.1%)	8 (12.3%)	3 (4.6%)	7 (5.1%)
Diet or dietary pattern	34 (20.6%)	11 (15.3%)	5 (7.7%)	11 (16.9%)	22 (16.1%)
Supplementation	30 (18.2%)	31 (43.1%)	49 (75.5%)	25 (38.5%)	53 (38.7%)
Complete formulae enteral or parenteral	2 (1.2%)	1 (1.4%)	0 (0%)	6 (9.2%)	3 (2.2%)
Nutrition education	84 (50.9%)	20 (27.8%)	3 (4.6%)	18 (27.7%)	49 (35.8%)
Breastfeeding	3 (1.8%)	1 (1.4%)	0 (0%)	1 (1.5%)	1 (0.7%)
Other	0 (0%)	0 (0%)	0 (0%)	0 (0%)	2 (1.5%)

Characteristics	USA (n = 165)	UK (n = 72)	Iran (n = 65)	China (n = 65)	Australia (n = 137)
C - Comparator					
Placebo	33 (20.0%)	27 (37.5%)	48 (73.8%)	23 (35.4%)	48 (35.0%)
Other intervention	63 (38.2%)	20 (27.8%)	9 (13.8%)	15 (23.1%)	26 (19.0%)
Usual care	52 (31.5%)	18 (25.0%)	5 (7.7%)	18 (27.7%)	50 (36.5%)
No control	17 (10.3%)	7 (9.7%)	3 (4.6%)	9 (13.8%)	13 (9.5%)
O - Primary Outcomes (most frequent)					
Clinical status	36 (21.8%)	14 (19.4%)	49 (75.4%)	20 (30.8%)	33 (24.1%)
Nutritional status	62 (37.6%)	11 (15.3%)	7 (10.8%)	13 (20.0%)	25 (18.2%)
Frequency or severity of disease	22 (13.3%)	15 (20.8%)	14 (21.5%)	20 (30.8%)	32 (23.4%)
Withdrawal, drop-out or adherence	13 (7.9%)	16 (22.2%)	3 (4.6%)	1 (1.5%)	8 (5.8%)
S - Study design					
Crossover	9 (5.5%)	5 (6.9%)	2 (3.1%)	0 (0%)	7 (5.1%)
Parallel	156 (94.5%)	67 (93.1%)	63 (96.9%)	65 (100%)	130 (94.9%)

**Characteristics**	**USA (n = 165)**	**UK (n = 72)**	**Iran (n = 65)**	**China (n = 65)**	**Australia (n = 137)**
Transparency and reproducibility practices					
Protocol register	137 (83.0%)	65 (90.3%)	64 (98.5%)	64 (98.5%)	132 (96.4%)
Conflicts of interest statement	108 (66.4%)	68 (94.4%)	64 (98.5%)	63 (96.9%)	130 (94.9%)
Funding statement	161 (97.6%)	71 (98.6%)	62 (95.4%)	62 (95.4%)	130 (94.9%)
Mention of SPIRIT	23 (13.9%)	22 (30.6%)	36 (55.4%)	23 (35.4%)	34 (24.8%)
Mention of TIDIeR	1 (0.6%)	6 (8.3%)	0 (0%)	0 (0%)	3 (2.2%)
Mention of CONSORT	35 (21.2%)	20 (27.8%)	13 (20.0%)	15 (23.1%)	56 (40.9%)

Supplementary Table 4. Absolute and relative frequency of PICOS categories and transparency and reproducibility principles used in protocols of nutrition- and diet-related RCT trials published 2012-2022 that focus on participants with cancer or cardiovascular disease.

	Cancer (n = 76)		Cardiovascular disease (n = 90)	
	n	%	n	%
**Population categories**				
Infant	0	0	1	1.1
Children	2	2.6	1	1.1
Adolescent	2	2.6	2	2.2
Adult	6	7.9	7	7.8
Adult and elderly	61	80.3	73	81.1
Elderly	1	1.3	7	7.8
Pregnant	0	0	1	1.1
Postmenopausal women	0	0	1	1.1
Families	3	3.9	1	1.1
Mother and infant	0	0	0	0
**Intervention type**				
Isolated	48	63.2	67	74.4
Combined with exercise	16	21.1	5	5.6
Combined with drug	3	3.9	1	1.1

Combined with other medical care	0	0	0	0
Part of lifestyle modification	8	10.5	7	7.8
Part of health programme	1	1.3	10	11.1
**Intervention categories**				
Food	3	3.9	12	13.3
Diet or dietary pattern	22	28.9	19	21.1
Supplementation	25	32.9	33	36.7
Complete formulae	11	14.5	2	2.2
Nutrition education	15	19.7	24	26.7
Breastfeeding	0	0	0	0
Other	0	0	0	0
**Comparator categories**				
Placebo	20	26.3	34	37.8
Other intervention	21	27.6	29	32.2
Usual care	32	42.1	23	25.6
No control	3	3.9	4	4.4
**Primary outcomes categories (the three most frequent)**				
Clinical status	11	14.5	39	43.3
Nutritional status	15	19.7	7	7.8
Frequency or severity of disease	19	25.0	22	24.4
**Study design**				

Crossover	1	1.3	5	5.6
Parallel	75	98.7	85	94.4
**Other information - disclosure of conflicts of interest**				
No reporting	7	9.2	12	13.3
Reporting of conflicts of interest	11	14.5	13	14.4
Reporting of no conflicts of interest	58	76.3	65	72.2
**Other information - disclosure of funding**				
No reporting	1	1.3	2	2.2
Reporting of funding	67	88.2	87	96.7
Reporting of no funding	8	10.5	1	1.1
**Other information - registry of protocol**				
Yes	69	90.8	88	97.8
No	7	9.2	2	2.2
